# Association between maternal polycystic ovarian syndrome undergoing assisted reproductive technology and pregnancy complications and neonatal outcomes: a systematic review and meta-analysis

**DOI:** 10.1186/s13048-023-01331-x

**Published:** 2024-01-06

**Authors:** Miaomiao Ban, Yifei Sun, Xiaojing Chen, Xiaoqian Zhou, Yiyuan Zhang, Linlin Cui

**Affiliations:** 1 Center for Reproductive Medicine, Cheeloo College of Medicine, Shandong University, Jinan, 250012 Shandong China; 2 Center for Reproductive Medicine, the Second Hospital, Cheeloo College of Medicine, Shandong University, Jinan, 250012 Shandong China; 3 Research Unit of Gametogenesis and Health of ART-Offspring, Chinese Academy of Medical Sciences, No.2021RU001), Jinan, 250012 Shandong China; 4 Key laboratory of Reproductive Endocrinology of Ministry of Education, Shandong University, Jinan, 250012 Shandong China; 5 Shandong Key Laboratory of Reproductive Medicine, Shandong Provincial Hospital, Shandong First Medical University, Jinan, 250012 China; 6 Shandong Provincial Clinical Research Center for Reproductive Health, Jinan, 250012 Shandong China; 7 Shandong Technology Innovation Center for Reproductive Health, Jinan, 250012 Shandong China; 8 National Research Center for Assisted Reproductive Technology and Reproductive Genetics, Shandong University, Jinan, 250012 Shandong China

**Keywords:** Polycystic ovarian syndrome, Assisted reproductive technology, Pregnancy complication, Neonatal outcome, Meta-analysis

## Abstract

**Background:**

Polycystic ovarian syndrome (PCOS) is recognized as the most prevalent endocrine disorder among women of reproductive age. While the utilization of assisted reproductive technology (ART) has resulted in favorable outcomes for infertility treatment in PCOS patients, the inherent pathophysiological features of the condition give rise to complications and consequences during pregnancy and delivery for both the mother and offspring. This study was to assess the correlation between maternal PCOS and various pregnancy complications and neonatal outcomes undergone ART.

**Methods:**

A systematic search was conducted on PubMed, EmBase, and the Cochrane Library to identify observational studies that investigated the association between PCOS and the risk of various pregnancy complications and neonatal outcomes, including gestational diabetes mellitus (GDM), hypertension in pregnancy (PIH), preeclampsia (PE), preterm birth, abortion, congenital malformations (CA), small for gestational age (SGA), large for gestational age (LGA), low birth weight (LBW), macrosomia, neonatal intensive care unit (NICU) admission and birth weight. Eligible studies were selected based on predetermined inclusion and exclusion criteria. The meta-analysis was conducted using Review Manager and Stata software, with odds ratios (ORs) or mean difference (MD), confidence intervals (CIs), and heterogeneity (I^2^) being calculated. The search was conducted up to March 2023.

**Results:**

A total of 33 studies with a combined sample size of 92,810 participants were identified. The findings indicate that PCOS is significantly associated with an increased risk of GDM (OR 1.51, 95% CI:1.17–1.94), PIH (OR 1.72, 95% CI:1.25–2.39), PE (OR 2.12, 95% CI:1.49–3.02), preterm birth (OR 1.29, 95% CI:1.21–1.39), and LBW (OR 1.29, 95% CI:1.14–1.47). In subgroup analyses, the risks of GDM (OR 1.80, 95% CI:1.23–2.62) and abortion (OR 1.41, 95% CI:1.08–1.84) were elevated in fresh embryo transferred (ET) subgroup, whereas elevated risk of PE (OR 1.82, 95% CI:1.17–2.83) and preterm birth (OR 1.31, 95% CI:1.21–1.42) was identified in frozen ET subgroup. Whatever with or without hyperandrogenism, patients with PCOS had a higher risk in preterm birth (OR 1.69, 95% CI: 1.31–2.18; OR 1.24, 95% CI:1.02–1.50) and abortion (OR 1.38, 95% CI:1.12–1.71; OR 1.23, 95% CI:1.06–1.43).

**Conclusion:**

Our findings suggest that individuals with PCOS undergone ART are at a notably elevated risk for experiencing pregnancy complications and unfavorable neonatal outcomes. Nevertheless, to establish a definitive association between PCOS and pregnancy-related outcomes, it is necessary to conduct extensive prospective, blinded cohort studies and effectively control for confounding variables.

**Supplementary Information:**

The online version contains supplementary material available at 10.1186/s13048-023-01331-x.

## Introduction

Polycystic ovarian syndrome (PCOS) is recognized as the most prevalent endocrine disorder among women of reproductive age, with an estimated prevalence ranging from 6 to 15%, depend on the diagnostic criteria employed [1, 2]. It is characterized by hyperandrogenism, anovulatory dysfunction, and polycystic ovaries [3], frequently accompanied by insulin resistance [4]. PCOS is a heterogeneous condition with a diverse range of phenotype, presenting a distinctive challenge to both patient care and medical research [2]. Furthermore, given that women diagnosed with PCOS faced reduced fertility potential regardless of ovulatory status, infertility is a typical outcome [5, 6], necessitating the use of assisted reproductive technology (ART) for conception. While the utilization of ART has resulted in favorable outcomes for infertility treatment in PCOS patients, the inherent pathophysiological features of the condition can give rise to complications and consequences during pregnancy and delivery for both the mother and offspring.

Currently, there exists mounting evidence indicating a heightened occurrence of pregnancy complications in women diagnosed with PCOS. Prior investigations have demonstrated the correlation between PCOS and unfavorable pregnancy complications and neonatal outcomes [7, 8]. Nevertheless, it has not been uniformly observed in other study [9]. To date, numerous systematic reviews and meta-analyses have been conducted on the association between PCOS and adverse pregnancy-related outcomes [10–12]. However, the heterogeneity of previous studies necessitates further examination of confounding variables and more comprehensive subgroup analyses. In light of recently published data, a meta-analysis is imperative to enhance the existing evidence regarding the correlation between PCOS and unfavorable outcomes following ART conception.

The significant worldwide prevalence of PCOS and its association with adverse pregnancy-related outcomes necessitates an expeditious clarification of its substantial role in the etiology of such outcomes. This would facilitate the development of interventions for women of childbearing age to mitigate the incidence of adverse neonatal outcomes attributed to PCOS. The objective of this systematic review and meta-analysis is to evaluate the correlation between PCOS and unfavorable pregnancy-related outcomes in the assisted reproduction population, and to offer suggestions for preventive medicine and public health.

## Materials and methods

This systematic review and meta-analysis adhered to the guidelines set forth by the Preferred Reporting Item for Systematic Reviews and Meta-analysis statement (PRISMA) [13]. We registered this meta-analysis in the International Prospective Register of Systematic Reviews (PROSPERO) with the registration number CRD42021282361. A pre-established protocol was developed and implemented. The Population/Income/Comparison/Outcome (PICO) question was formulated as follows: Are infertile ART patients with PCOS at increased risk of gestational diabetes mellitus (GDM), pregnancy-induced hypertension (PIH), preeclampsia (PE), preterm birth, abortion, congenital malformations (CA), small for gestational age (SGA), large for gestational age (LGA), low birth weight (LBW), macrosomia, neonatal intensive care unit (NICU) admission and/or birth weight in comparison with controls without PCOS diagnosis?

### Data sources and search strategy

In order to identify research of high caliber, a comprehensive search was conducted from the inception of the databases until March 2023, utilizing three electronic sources, namely Pubmed, Embase, and Cochrane Library. The search strategy was formulated by combining relevant search terms:[‘Polycystic Ovary Syndrome’ (MeSH) AND ‘Delivery, Obstetric’ OR ‘Labor, Obstetric’ OR ‘pregnancy complications’ OR ‘Obstetric Labor Complications’ OR ‘Diabetes, Gestational’ OR ‘Hypertension, Pregnancy-Induced’ OR ‘Pre-Eclampsia’ OR ‘Cesarean Section’ OR ‘Premature Birth’ OR ‘Infant, Low Birth Weight’ OR ‘Infant, Small for Gestational Age Pregnant Women’ OR ‘Gravidity’ OR ‘Fetal Growth Retardation’ OR ‘Infant Health’ OR ‘Perinatal Death’ OR ‘Child Health’ OR ‘Infant, Newborn’ OR ‘Autism Spectrum Disorder’]. In addition to conducting a manual search of the reference lists of pertinent original and review articles, we identified further eligible studies. The comprehensive search strategy is available in Supplementary Table 1. All relevant literature was exported to the reference manager software (ENDNOTE R¸X9, Bld 7212, Thomson Reuters) and duplicates were eliminated. We also conducted manual evaluations to ascertain the uniqueness of articles.

### Study selection and eligibility criteria

Two authors (M. B, Y. S) independently conducted the search strategy and identified studies using standardized methods. Any discrepancies were addressed and resolved by the group through consensus. Subsequently, the remaining articles were thoroughly examined, and eligible articles were selected based on the following inclusion criteria: [1] an observational study design, whether prospective or retrospective; [2] researches needed to involve women with PCOS who had undergone ART, the control group consisted of women without PCOS diagnosis who had undergone ART. The control group was subject to restrictions that excluded any diseases that could potentially impact pregnancy-related outcomes, and the specific characteristics of the control group are detailed in Supplementary Table 2; [3] researches should investigate the relationship between PCOS and adverse outcomes during pregnancy, including those affecting the fetus and neonate; [4] the calculation of odds ratios (OR) and their corresponding 95% confidence intervals (CIs) was possible, or alternatively, the number of outcomes and sample size in each group could be used for comparison purposes. The exclusion criteria comprised of four categories: [1] incomplete data, which encompassed unavailable data, unclear or inappropriate definition of cases, and unadjusted confounders; [2] editorials, reviews, and letters to the editor; [3] animal research; and [4] languages other than English.

The diagnosis of PCOS was based on the 2003 Rotterdam diagnostic criteria, which at least two of three following criteria were present: oligomenorrhea and/or anovulation; clinical and/or biochemical signs of hyperandrogenism; and polycystic ovaries on ultrasound scanning [14]. The ART treatments included controlled ovarian hyperstimulation, classical IVF or ICSI, endometrial preparation, embryo culture, and fresh or frozen embryo transfer.

### Data extraction and quality assessment

In accordance with the eligibility criteria, two authors conducted an independent assessment of the titles, abstracts, and full text for inclusion. Any uncertainties regarding inclusion were resolved through discussion with a third reviewer. Extracted information of all eligible studies included title, author names, year of publication, country, study design, sample size, participants’ characteristics (such as race, age, and BMI), information of control group, primary outcomes (prevalence of GDM/PIH/PE/abortion/preterm birth/CA/SGA/LGA/LBW/macrosomia/NICU admission/birth weight expressed by the number of cases, calculated OR and its 95% CI), and adjusted confounders. All information was entered into a researcher-developed data extraction form.

The quality of eligible studies was assessed using the Newcastle-Ottawa Quality Assessment Scale (NOS), a validated tool recommended by the Cochrane Working Group for assessing the risk of bias in non-randomized studies [15]. The revised scale proposed by Barry et al. [16] adapted the entry “determination of diagnosis” to “determination of exposure” to make the NOS applicable to cross-sectional studies. The NOS checklist comprises three quality parameters: sample selection, comparability, and exposure. Each parameter includes questions with scoring options of one or two points, depending on whether the criteria are met. Based on the NOS scores, studies were categorized as low quality (0–3 points), medium quality (4–6 points), or high quality (7–9 points). The disparities were reconciled by M.B. and Y.S., the two reviewers by discourse and supplementary feedback from researchers who were not affiliated with the authorship.

### Statistical analysis

All statistical analyses were performed using the Review Manager (version 5.3, The Cochrane Collaboration, Copenhagen, Denmark) and Stata (version 12, StataCorp, College Station, TX). Q test and I^2^ test were used to assess the heterogeneity across studies [17]. In the event that the article exhibited homogeneity, as indicated by *P* > 0.05 or I^2^ < 50%, the fixed effect model, specifically the Mantel-Haenszel method, was employed to evaluate the additional uncertainty linking PCOS and adverse pregnancy outcomes. Conversely, if homogeneity was not observed, the random-effect model, specifically the DerSimonian-Laird method, was utilized. To determine the possibility of publication bias, a funnel plot was constructed. Subgroup analyses were conducted to investigate the potential association between ART interventions and overall OR values. Graphical and statistical assessments of publication bias were performed using Egger’s linear regression test, with a significance level of *P* < 0.10 [18]. Finally, sensitivity analysis was performed by excluding one study per round using the meta algorithm in Stata.

## Result

### Search results

**Fig. 1:**
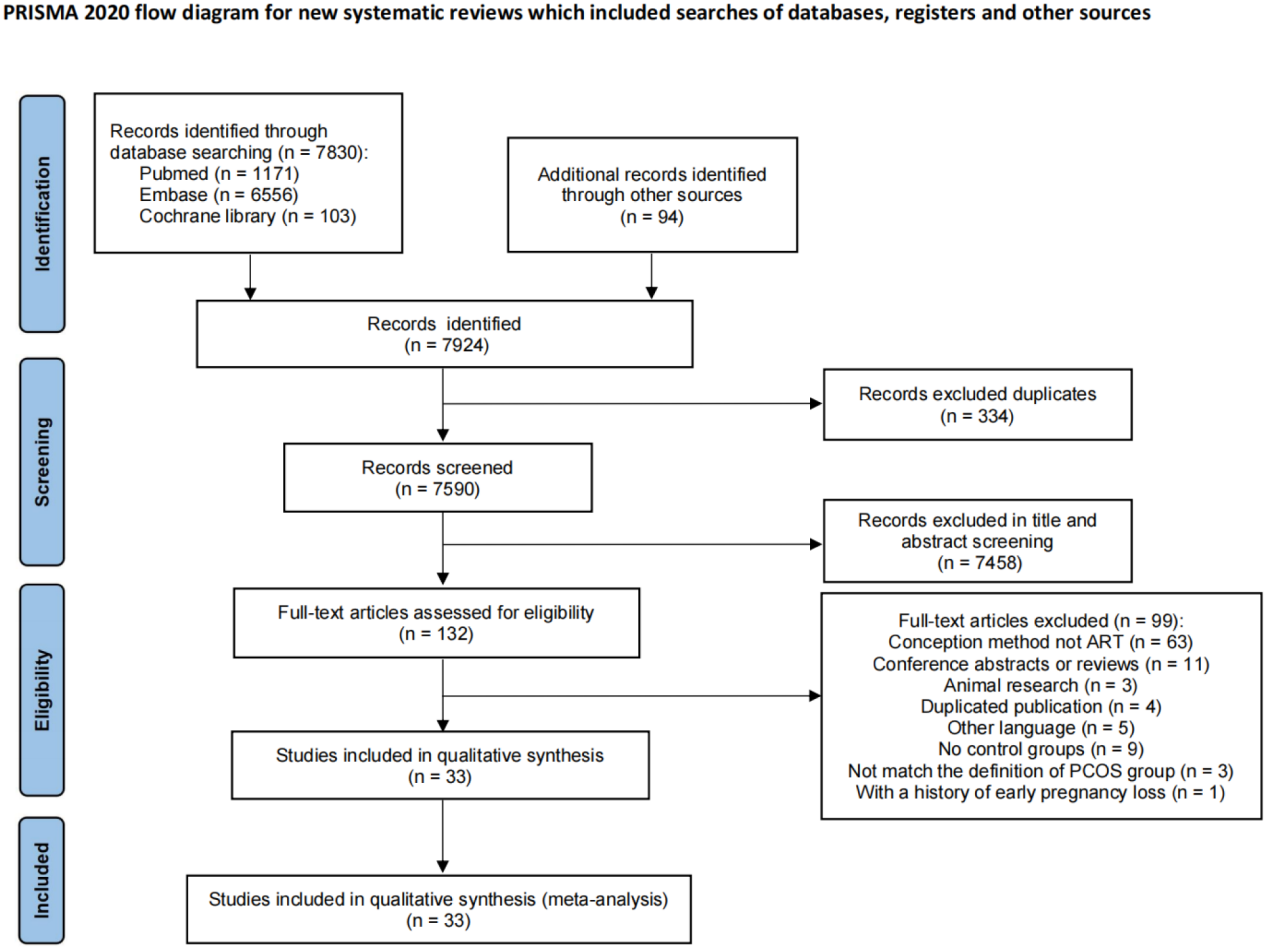
Flowchart of the study selection process for the present meta-analysis

The flowchart (Fig. 1) displays the search results. The electronic search produced 7830 articles, while 94 articles were obtained from the reference lists of relevant articles, reviews, and papers. After excluding 334 studies due to duplication and 7466 records based on the initial scan of titles and abstracts, 132 studies remained for a thorough comprehensive assessment review. Subsequently, 63 studies were excluded due to the absence of ART-assisted conception in all patients, while 11 were excluded for being conference articles or reviews. Additionally, 3 studies were excluded for being animal research, 4 for being duplicated publications, and 5 for being written in languages other than English. Furthermore, 9 studies lacked control groups, 3 study did not meet the definition of the PCOS group, and 1 study had a history of early pregnancy loss, resulting in their exclusion from the analysis. Finally, 33 observational studies were included in this meta-analysis [19–51]. And 14 were published in the last three years [38–51]. For the previous article, if it detailed the diagnostic and treatment methods that meet the standards, it was also included [19–29].

### Characteristics of the included studies

The 33 eligible studies reported 92,810 pregnant women in 4 prospective cohort studies [21, 32, 37, 49], 23 retrospective cohort studies [20, 22–25, 27–30, 33–36, 38, 40, 41, 43, 44, 46–48, 50, 51], 5 case-control studies [19, 26, 31, 39, 42], and 1 cross-sectional study [45]. All of the studies conducted in this analysis aimed to compare the prevalence of pregnancy complications and neonatal outcomes between women diagnosed with PCOS and the control group in the assisted reproduction population. The results of the analysis revealed that, with regards to pregnancy complications, 15 studies investigated GDM, 15 studies investigated PIH, 8 studies investigated PE, 18 studies investigated preterm birth, and 26 studies investigated abortion. In terms of neonatal outcomes, 6 studies investigated CA, 8 studies investigated SGA, 7 studies investigated LGA, 9 studies investigated LBW, 8 studies investigated macrosomia, 5 studies investigated NICU admission, and 7 studies investigated birth weight. 14 studies were conducted in China [33–35, 37, 38, 40–43, 46–48, 50, 51], 2 in America [23, 29], 2 in Japan [22, 24], 2 in Sweden [26, 44], 2 in Canada [19, 36] and the remaining 8 in Israel [20], Britain [21], Italy [25], Netherlands [28], Slovenia [39], Norway [27], Korea [31], India [49], Turkey [45], Finland [30] and Iran [32]. Characteristics of all studies are displayed in Supplemental Table 2.

### Comparison in pregnancy complications

**Fig. 2:**
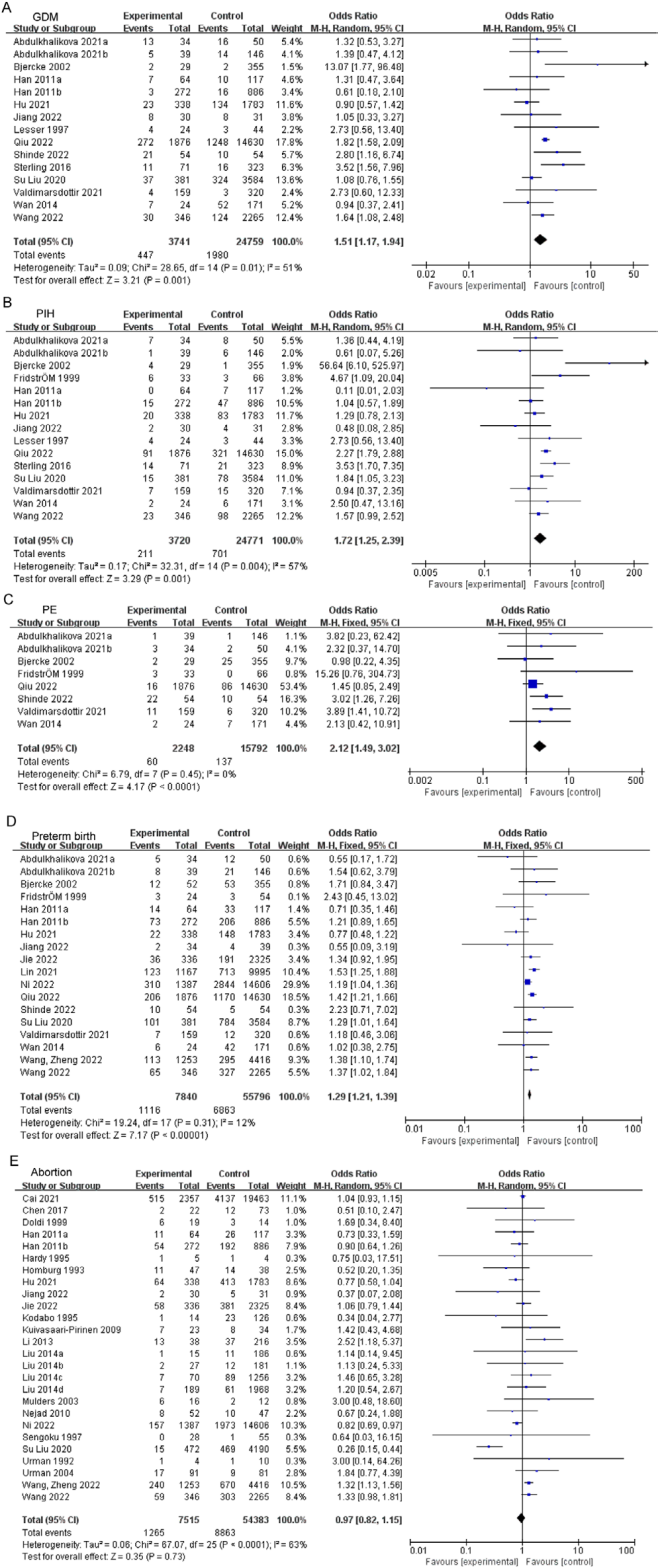
Forest plot displayed odds of GDM (**A**), PIH (**B**), PE (**C**), preterm birth (**D**), and abortion (**E**) in PCOS patients versus controls. GDM, gestational diabetes mellitus; PIH, hypertension in pregnancy; PE, preeclampsia

**Fig. 3:**
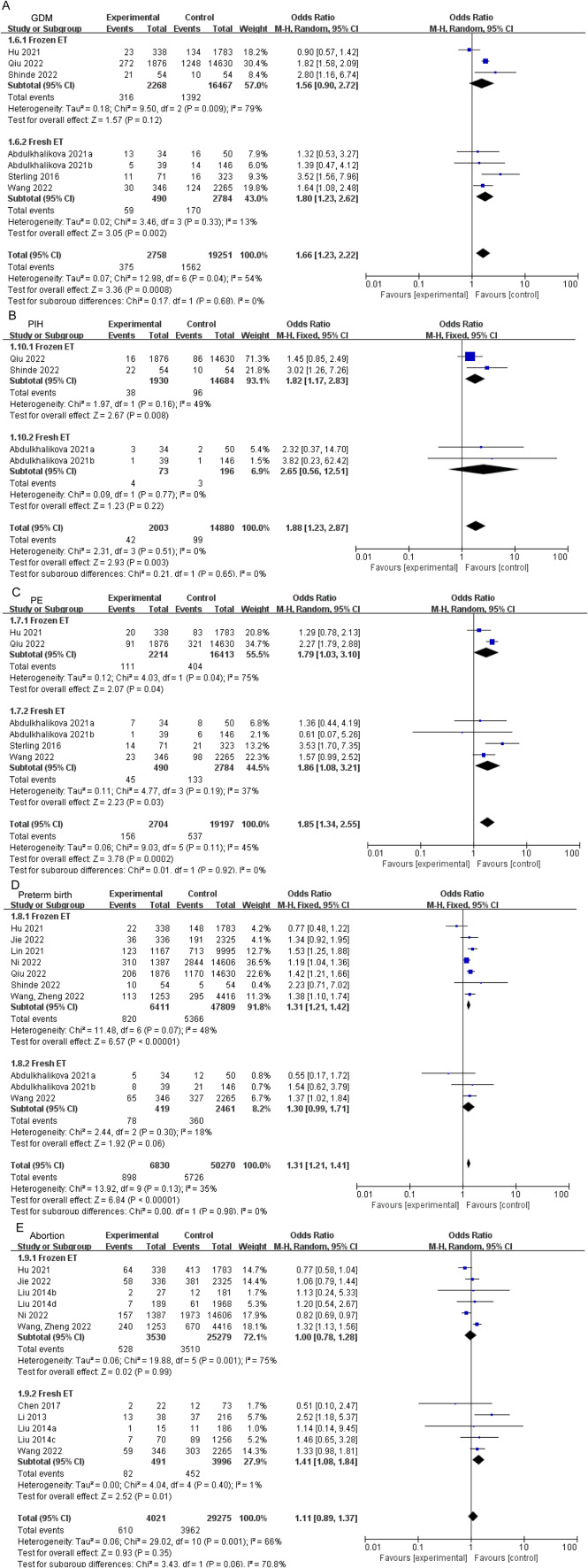
Subgroup analyses in transfer method for pregnancy complications: GDM (**A**), PIH (**B**), PE (**C**), preterm birth (**D**), and abortion (**E**). GDM, gestational diabetes mellitus; PIH, hypertension in pregnancy; PE, preeclampsia

**Fig. 4:**
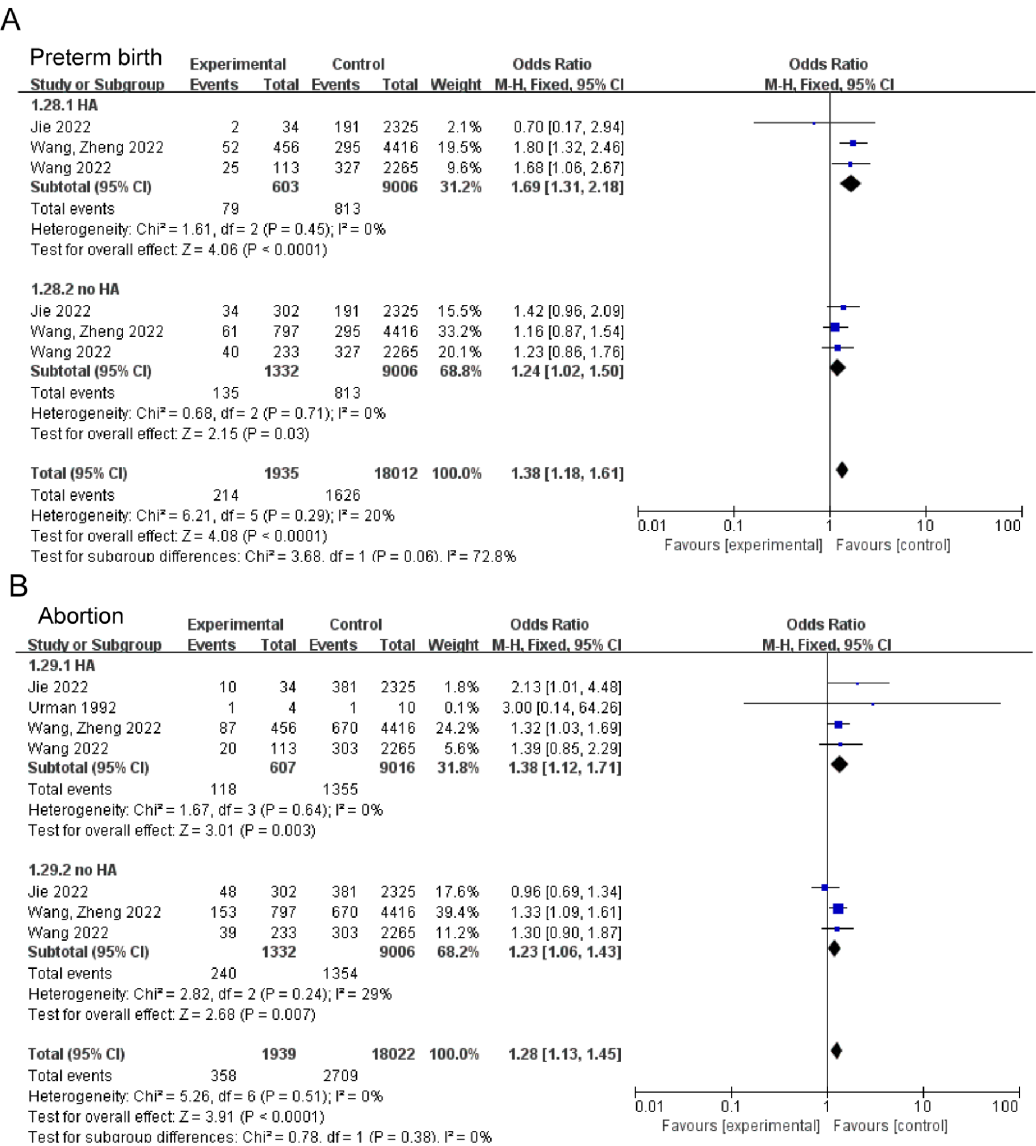
Subgroup analyses in with or without hyperandrogenism for pregnancy complications: preterm birth (**A**), and abortion (**B**)

According to meta-analysis, PCOS patients exhibited increased risk of GDM (OR: 1.51, 95% CI: 1.17–1.94, *P* = 0.001; I^2^ = 51%, *P*_Q_ = 0.01, Fig. 2A), PIH (OR: 1.72, 95% CI: 1.25–2.39, *P* = 0.001; I^2^ = 57%, *P*_Q_ = 0.004, Fig. 2B), and PE (OR: 2.12, 95% CI: 1.49–3.02, *P* < 0.0001; I^2^ = 0%, *P*_Q_ = 0.45, Fig. 2C) when compared to the control group. In addition, women with PCOS also showed increased risk of preterm birth (OR: 1.29, 95% CI: 1.21–1.39, *P* < 0.00001; I^2^ = 12%, *P*_Q_ = 0.31, Fig. 2D). However, there was no remarkable difference of prevalence in abortion (Fig. 2E). Subgroups with increased risks of pregnancy complications were listed in Supplemental Table 4. Caucasian patients with PCOS exhibited elevated risks of GDM, PIH, and PE, while Indian patients with PCOS demonstrated elevated risks of GDM and PE. Conversely, East Asian patients with PCOS were found to have increased risk of PIH and preterm birth. In subgroup analyses, the risks of GDM (OR:1.80, 95% CI:1.23–2.62, *P* = 0.002, Fig. 3A) and abortion (OR: 1.41, 95% CI: 1.08–1.84, *P* = 0.01, Fig. 3D) were elevated in fresh ET patients with PCOS, whereas elevated risk of PE (OR: 1.82, 95% CI: 1.17–2.83, *P* = 0.008, Fig. 3C) and preterm birth (OR: 1.31, 95% CI: 1.21–1.42, *P* < 0.00001, Fig. 3D) was identified in frozen ET. Both transfer methods increased risk of PIH in patients with PCOS compared to controls. Whatever with or without hyperandrogenism, patients with PCOS had a higher risk in preterm birth (OR: 1.69, 95% CI: 1.31–2.18, *P* < 0.0001, Fig. 4A; OR: 1.24, 95% CI: 1.02–1.50, *P* = 0.03, Fig. 4A) and abortion (OR: 1.38, 95% CI: 1.12–1.71, *P* = 0.003, Fig. 4B; OR: 1.23, 95% CI: 1.06–1.43, *P* = 0.007, Fig. 4B). The comprehensive findings of the subgroup analysis can be observed in Supplementary Tables 5–9.

### Comparison in neonatal outcomes

**Fig. 5:**
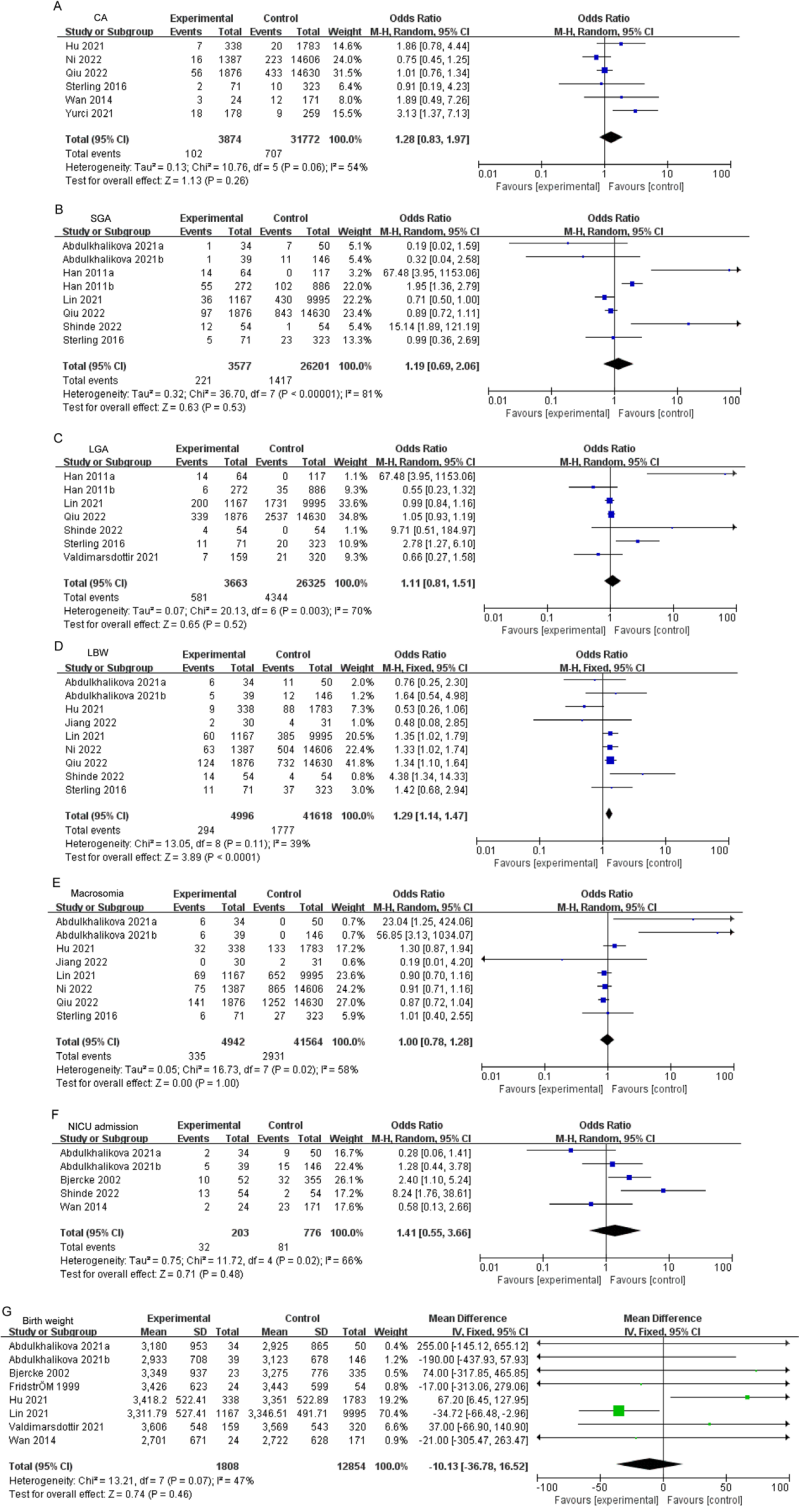
Forest plot displayed odds of CA (**A**), SGA (**B**), LGA (**C**), LBW (**D**), macrosomia (**E**), NICU admission (**F**) and birth weight (**G**) in PCOS patients versus controls. CA, congenital malformations; SGA, small for gestational age; LGA, large for gestational age; LBW, low birth weight; NICU admission, neonatal intensive care unit admission

It was observed that individuals with PCOS displayed a heightened prevalence to LBW comparison to the control group (OR: 1.29, 95% CI: 1.14–1.47, *P* < 0.0001; I^2^ = 39%, *P*_Q_ = 0.11, Fig. 5D). However, there was no significant difference in the prevalence of CA, SGA, LGA, macrosomia, NICU admission and birth weight. The subgroups exhibiting increased risks of neonatal outcomes were documented in Supplemental Table 4. In subgroup analyses, elevated risk of LBW was identified in East Asian (OR: 1.27, 95% CI: 1.11–1.45, *P* = 0.0005, Supplementary Table 13). Detailed results of the subgroup analysis are shown in Supplementary Tables 10–16.

### Sensitivity analysis and publication bias

The findings of the sensitivity analysis and assessment of publication bias are presented in Supplementary Table 17. Sensitivity analyses were conducted to assess the impact of heterogeneity among the included studies on the overall risk estimate. The sensitivity analysis reveals an influence on the pooled Mean Difference and 95% CI of birth weight when Lin’s study was omitted (Mean Difference: 48.43, 95% CI: -0.58-97.44). Publication bias was assessed using Egger’s linear regression test for each outcome, and no evidence of publication bias was observed across the studies included in this meta-analysis.

## Discussion

The present investigation utilized observational studies to examine the potential associations between PCOS and the likelihood of adverse outcomes during pregnancy, fetal and neonatal health following ART. Our meta-analysis revealed that expectant mothers with PCOS exhibited a significantly greater risk of GDM, PIH, PE, preterm birth and LBW compared to the control group. Furthermore, a marginally significant decrease in birth weight was observed in PCOS group. However, there was no significant difference between pregnancies with and without PCOS diagnosis in terms of abortion, CA, LGA, SGA, macrosomia, NICU admission, and birth weight.

Several previous systematic reviews and meta-analyses reported similar conclusions regarding the potential adverse impact of PCOS on pregnancy, which the information of seven published reviews was exhibited in Supplementary Table 18. Specifically, Hai-Feng Yu et al. [52] found that women with PCOS face an elevated risk of GDM, PIH, PE, preterm birth and abortion during pregnancy. Similarly, Jun Z Qin et al. [53] observed a significantly higher incidence of GDM, PIH, PE, and preterm birth in pregnant women with PCOS compared to those without the condition. Furthermore, Kjerulff et al. [11] discovered that PCOS during pregnancy was linked to an increased likelihood of maternal complications, including GDM, PIH, PE, and preterm birth. Previous meta-analysis on the subject of PCOS and pregnancy outcomes has demonstrated a significant degree of heterogeneity [54], which may be attributed to the influence of diverse confounding factors. Our results are similar to those of the previous articles, but we have updated articles and made the ranking criteria more stringent.

The pathophysiological mechanisms underlying these relationships remain inadequately elucidated. Oocyte competence abnormalities may be one of the mechanisms [55]. Patients diagnosed with PCOS exhibit deficiencies in gene expression related to oocyte meiosis and early embryonic development, which impede oocyte development and embryo quality [55, 56]. Additionally, the endocrine milieu contributes to reduced endometrial tolerance, heightened luteinizing hormone (LH) levels, a substantially elevated risk of miscarriage, and a decreased rate of conception [57]. Antimullerian hormone (AMH) is nowadays considered a novel biomarker for fetal and placental health [58, 59]. Elevated AMH levels leads to the changes of maternal brain, ovaries, and placenta which may cause adverse pregnancy outcomes [60]. During pregnancy, the synthesis of placental prolactin, estrogen, progesterone, placental insulinase, and maternal adrenocorticotropic hormone production all exhibit antagonistic effects against insulin, ultimately leading to decreased insulin sensitivity in the body [61]. These pathophysiological characteristics may result in adverse pregnancy complications and neonatal outcomes in women with PCOS. The co-occurrence of hyperandrogenism and insulin resistance in PCOS patients has been suggested to contribute to pregnancy complications [27]. This phenomenon may arise through either direct augmentation of androgen production or indirect reduction of sex hormone binding globulin (SHBG) production [62]. Furthermore, prior research has demonstrated a strong correlation between preconception SHBG levels in women with PCOS and the subsequent development of GDM [63]. In the context of PCOS, hyperandrogenism is intricately linked to the occurrence and extent of microscopic alterations in early trophoblast invasion and placentation [64]. The proposition has been put forth that the mediation of hemodynamic changes leading to the onset of PE may be attributed to free testosterone, which induces a state of sympathetic and vascular hyperactivity [65]. Furthermore, elevated levels of androgens may impede maternal energy homeostasis and nutrient transport through the placenta, as well as directly impact fetal growth, thereby affecting neonatal weight [66, 67].

Another explanation was that PCOS had also been characterized by a similar state of chronic low-grade inflammation [68], thereby increasing the production of specific cytokines and chemokines such as tumor necrosis factor-α (TNF-α), interleukin-6 (IL-6) and interleukin-1 (IL-1), adhesion molecules and endothelial dysfunction, follicle inhibitor as well as c-reactive protein [69–71]. The proinflammatory mediators IL-1 and TNF-α can directly induce the expression of cyclooxygenase 2 (COX-2) in the amniotic and metaphase membranes, thereby promoting the production of PGE2. Simultaneously, the upregulation of matrix metalloproteinases (MMPs) in the amniotic chorion, meconium, and cervix results in the degradation of the extracellular matrix of the fetal membrane and cervix, ultimately contributing to unfavorable neonatal outcomes [72]. Several studies have reported high levels of inflammatory cytokines in amniotic fluid of premature women [73, 74], suggesting that potential inflammatory mediators linked to PCOS may also precipitate premature birth. In addition, inflammation and immune damage, prenatal and postnatal hormonal abnormalities, and metabolic changes may cause endometrial dysfunction, which predisposes to poor pregnancy outcomes [75].

The present meta-analysis contributes to the provision of dependable information regarding the prevalence of obstetric and neonatal outcomes subsequent to ART in women diagnosed with PCOS. Our study possesses some distinctive advantages. Firstly, it is founded on a more extensive and current database, incorporating a greater number of observations and a more comprehensive subgroup analysis. Secondly, our study stratifies the articles into distinct subgroups based on the embryo transfer methods, thereby partially elucidating the origin of heterogeneity. Thirdly, our meta-analysis includes substantial number of qualified studies. The ample quantity of studies enhances the statistical potency and furnishes dependable and accurate estimations of the outcomes. In our study, we have adopted a more scientific definition of the control group as women without PCOS diagnosis, which makes our conclusions more medically compelling. Nevertheless, there exist certain limitations to the current research. Initially, it is worth noting that effect estimates derived from individual studies are subject to varying adjusting factors that may significantly contribute to the emergence of adverse pregnancy, fetal, and neonatal outcomes. Secondly, since all the studies incorporated in this analysis are published articles, publication bias is an inescapable concern. Furthermore, the limited number of studies that stratify different PCOS phenotypes does not provide a comprehensive understanding of how the prevalence of pregnancy and neonatal complications varies with the phenotype of PCOS. Finally, prospective data to establish causality included in our study was less.

The present study holds significant implications for contemporary clinical practice. Given that PCOS is a modifiable risk factor for unfavorable pregnancy outcomes, we recommend that medical practitioners continue to regard patients with PCOS as a high-risk population and provide close monitoring for the emergence of adverse pregnancy complications or neonatal outcomes. Such measures may contribute to mitigating the incidence of unfavorable neonatal outcomes in pregnant women. The findings of this study also raise concerns for women undergoing ART for PCOS. Therefore, further research with large sample sizes in randomized controlled trials is warranted.

## Conclusion

In conclusion, women with PCOS are at increased risk for poor pregnancy and neonatal complications in the assisted reproduction population. This knowledge is of utmost importance in the clinical management of pregnancy in patients with this condition. It is recommended that these women are made aware of the potential risks associated with their pregnancies and receive comprehensive monitoring, attention, and screening for these complications throughout the course of their pregnancy and delivery. Additional research is required to enhance the management of pregnancy in women afflicted with PCOS, with a focus on investigating the significance of glycemic control, hormonal status modulation, lifestyle modifications, and pharmacotherapy.

### Electronic supplementary material

Below is the link to the electronic supplementary material.

Supplementary Material 1

## Data Availability

The datasets and materials in this study are available from the corresponding author on reasonable request.
