# Supplementary material for: Association between maternal polycystic ovarian syndrome undergoing assisted reproductive technology and pregnancy complications and neonatal outcomes: a systematic review and meta-analysis
Source: J Ovarian Res. 2024 Jan 6;17:6. doi: 10.1186/s13048-023-01331-x (PMC10770902; doi:10.1186/s13048-023-01331-x)
Supplement: Supplementary file 1 — Supplementary Material 1 [file 13048_2023_1331_MOESM1_ESM.docx]

***Supplementary Materials***

Supplemental Table 1. Search strategy and selected keywords.

Supplemental Table 2. Characters of included studies.

Supplemental Table 3. Assessment of risk of bias based on Newcastle-Ottawa Scale.

Supplemental Table 4. Subgroups with pregnancy complications and neonatal outcomes.

Supplemental Table 5. Subgroup analyses for GDM.

Supplemental Table 6. Subgroup analyses for PIH.

Supplemental Table 7. Subgroup analyses for PE.

Supplemental Table 8. Subgroup analyses for preterm birth.

Supplemental Table 9. Subgroup analyses for abortion.

Supplemental Table 10. Subgroup analyses for CA.

Supplemental Table 11. Subgroup analyses for SGA.

Supplemental Table 12. Subgroup analyses for LGA.

Supplemental Table 13. Subgroup analyses for LBW.

Supplemental Table 14. Subgroup analyses for macrosomia.

Supplemental Table 15. Subgroup analyses for NICU admission.

Supplemental Table 16. Subgroup analyses for birth weight.

Supplemental Table 17. Sensitive analyses and publication bias for pregnancy complications and neonatal outcomes.

Supplementary Table 18. Information from published meta-analyses on pregnancy complications in women with PCOS.

Supplementary Table 1. Search strategy and selected keywords

Database: PubMed:

Search Strategy:窗体顶端

--------------------------------------------------------------------------------

(((((((((((((((((Polycystic Ovary Syndrome) OR Ovary Syndrome, Polycystic) OR Syndrome, Polycystic Ovary) OR Stein-Leventhal Syndrome) OR Stein Leventhal Syndrome) OR Syndrome, Stein-Leventhal) OR Sclerocystic Ovarian Degeneration) OR Ovarian Degeneration, Sclerocystic) OR Sclerocystic Ovary Syndrome) OR Polycystic Ovarian Syndrome) OR Ovarian Syndrome, Polycystic) OR Polycystic Ovary Syndrome 1) OR Sclerocystic Ovaries) OR Ovary, Sclerocystic) OR Sclerocystic Ovary AND (((((((((((((((((((((((((((((((((((((((((((((((((((((((((((((((((((((((((((((((((((((((((((((((((((((((((((((((((((((((((((((((((((((((((((((((((((((((((((((((((((((((((((((((((((((((Pregnancy) OR Pregnancies) OR Gestation) OR Gravidity) OR Gravidities) OR Delivery, Obstetric) OR Deliveries, Obstetric) OR Obstetric Deliveries) OR Obstetric Delivery) OR Labor, Obstetric) OR Obstetric Labor) OR pregnancy complications) OR Complication, Pregnancy) OR Pregnancy Complication) OR Complications, Pregnancy) OR Obstetric Labor Complications) OR Complication, Obstetric Labor) OR Complications, Obstetric Labor) OR Labor Complication, Obstetric) OR Labor Complications, Obstetric) OR Obstetric Labor Complication) OR Labor Complications) OR Complication, Labor) OR Labor Complication) OR Complications, Labor) OR Diabetes, Gestational) OR Diabetes, Pregnancy-Induced) OR Diabetes, Pregnancy Induced) OR Pregnancy-Induced Diabetes) OR Gestational Diabetes) OR Diabetes Mellitus, Gestational) OR Gestational Diabetes Mellitus) OR Hypertension, Pregnancy-Induced) OR Hypertension, Pregnancy Induced) OR Pregnancy-Induced Hypertension) OR Pregnancy Induced Hypertension) OR Hypertensions, Pregnancy Induced) OR Induced Hypertension, Pregnancy) OR Induced Hypertensions, Pregnancy) OR Gestational Hypertension) OR Hypertension, Gestational) OR Transient Hypertension, Pregnancy) OR Hypertension, Pregnancy Transient) OR Pregnancy Transient Hypertension) OR Pre-Eclampsia) OR Pre Eclampsia) OR Preeclampsia) OR Pregnancy Toxemias) OR Pregnancy Toxemia) OR Toxemia, Pregnancy) OR Edema-Proteinuria-Hypertension) OR Gestosis) OR Edema Proteinuria Hypertension Gestosis) OR Gestosis, Edema-Proteinuria-Hypertension) OR Hypertension-Edema-Proteinuria Gestosis) OR Gestosis, Hypertension-Edema-Proteinuria) OR Hypertension Edema Proteinuria Gestosis) OR Toxemia Of Pregnancy) OR Of Pregnancies, Toxemia) OR Of Pregnancy, Toxemia) OR Pregnancies, Toxemia Of) OR Pregnancy, Toxemia Of) OR Toxemia Of Pregnancies) OR EPH Complex) OR EPH Toxemias) OR EPH Toxemia) OR Toxemia, EPH) OR Toxemias, EPH) OR EPH Gestosis) OR Gestosis, EPH) OR Toxemias, Pregnancy) OR Preeclampsia Eclampsia 1) OR 1, Preeclampsia Eclampsia) OR 1s, Preeclampsia Eclampsia) OR Eclampsia 1, Preeclampsia) OR Eclampsia 1s, Preeclampsia) OR Preeclampsia Eclampsia 1s) OR Proteinuria-Edema-Hypertension Gestosis) OR Gestosis, Proteinuria-Edema-Hypertension) OR Proteinuria Edema Hypertension Gestosis) OR Cesarean Section) OR Cesarean Sections) OR Delivery, Abdominal) OR Abdominal Deliveries) OR Deliveries, Abdominal) OR Caesarean Section) OR Caesarean Sections) OR Abdominal Delivery) OR C-Section (OB)) OR C Section (OB)) OR C-Sections (OB)) OR Postcesarean Section) OR Premature Birth) OR Birth, Premature) OR Births, Premature) OR Premature Births) OR Preterm Birth) OR Birth, Preterm) OR Births, Preterm) OR Preterm Births) OR Infant, Low Birth Weight) OR Low-Birth-Weight Infant) OR Infant, Low-Birth-Weight) OR Infants, Low-Birth-Weight) OR Low Birth Weight Infant) OR Low-Birth-Weight Infants) OR Low Birth Weight) OR Birth Weight, Low) OR Birth Weights, Low) OR Low Birth Weights) OR Infant, Small for Gestational Age) OR Pregnant Women) OR Pregnant Woman) OR Woman, Pregnant) OR Women, Pregnant) OR Gravidity) OR Gravidities) OR Nulligravidity) OR Nulligravidities) OR Primigravidity) OR Primigravidities) OR Multigravidity) OR Multigravidities) OR Fetal Growth Retardation) OR Intrauterine Growth Retardation) OR Growth Retardation, Intrauterine) OR Intrauterine Growth Restriction) OR Fetal Growth Restriction) OR Infant Health) OR Health, Infant) OR Baby Health) OR Health, Baby) OR Newborn Health) OR Health, Newborn) OR Neonatal Health) OR Health, Neonatal) OR Health of Newborn Infants) OR Newborn Infant Health) OR Health, Newborn Infant) OR Infant Health, Newborn) OR Health of the Newborn Infant) OR Perinatal Death) OR Death, Perinatal) OR Deaths, Perinatal) OR Perinatal Deaths) OR Neonatal Death) OR Death, Neonatal) OR Deaths, Neonatal) OR Neonatal Deaths) OR Child Health) OR Health, Child) OR Childrens Health) OR Health, Childrens) OR Children's Health) OR Health, Children's) OR Child Well Being) OR Well Being, Child) OR Child Well-Being) OR Well-Being, Child) OR Child Wellbeing) OR Wellbeing, Child) OR Caesarean section) OR Cesarean Sections) OR Delivery, Abdominal) OR Abdominal Deliveries) OR Deliveries, Abdominal) OR Caesarean Section) OR Caesarean Sections) OR Abdominal Delivery) OR C-Section (OB)) OR C Section (OB)) OR C-Sections (OB)) OR Postcesarean Section) OR Pregnancy, Multiple) OR Multiple Pregnancy) OR Multiple Pregnancies) OR Pregnancies, Multiple) OR operative vaginal delivery) OR large-for-gestational-age infants) OR maternal complications) OR neonatal complications) OR reduced fetal growth) OR infantile catch-up in weight)))) NOT (((((((((Meta-Analysis as Topic) OR Data Pooling) OR Data Poolings) OR Overviews, Clinical Trial) OR Clinical Trial Overviews) OR Clinical Trial Overview) OR Overview, Clinical Trial) OR Meta-Analysis) OR Meta Analysis)

Database: Embase:

Search Strategy:窗体顶端

--------------------------------------------------------------------------------

#198 #17 AND #197

#197 #18 OR #19 OR #20 OR #21 OR #22 OR #23 OR #24 OR #25 OR #26 OR #27 OR #28 OR #29 OR #30 OR #31 OR #32 OR #33 OR #34 OR #35 OR #36 OR #37 OR #38 OR #39 OR #40 OR #41 OR #42 OR #43 OR #44 OR #45 OR #46 OR #47 OR #48 OR #49 OR #50 OR #51 OR #52 OR #53 OR #54 OR #55 OR #56 OR #57 OR #58 OR #59 OR #60 OR #61 OR #62 OR #63 OR #64 OR #65 OR #66 OR #67 OR #68 OR #69 OR #70 OR #71 OR #72 OR #73 OR #74 OR #75 OR #76 OR #77 OR #78 OR #79 OR #80 OR #81 OR #82 OR #83 OR #84 OR #85 OR #86 OR #87 OR #88 OR #89 OR #90 OR #91 OR #92 OR #93 OR #94 OR #95 OR #96 OR #97 OR #98 OR #99 OR #100 OR #101 OR #102 OR #103 OR #104 OR #105 OR #106 OR #107 OR #108 OR #109 OR #110 OR #111 OR #112 OR #113 OR #114 OR #115 OR #116 OR #117 OR #118 OR #119 OR #120 OR #121 OR #122 OR #123 OR #124 OR #125 OR #126 OR #127 OR #128 OR #129 OR #130 OR #131 OR #132 OR #133 OR #134 OR #135 OR #136 OR #137 OR #138 OR #139 OR #140 OR #141 OR #142 OR #143 OR #144 OR #145 OR #146 OR #147 OR #148 OR #149 OR #150 OR #151 OR #152 OR #153 OR #154 OR #155 OR #156 OR #157 OR #158 OR #159 OR #160 OR #161 OR #162 OR #163 OR #164 OR #165 OR #166 OR #167 OR #168 OR #169 OR #170 OR #171 OR #172 OR #173 OR #174 OR #175 OR #176 OR #177 OR #178 OR #179 OR #180 OR #181 OR #182 OR #183 OR #184 OR #185 OR #186 OR #187 OR #188 OR #189 OR #190 OR #191 OR #192 OR #193 OR #194 OR #195 OR #196 1,762,233

#196 'disorder, autistic spectrum':ti,ab 24

#195 'autistic spectrum disorders':ti,ab 1,120

#194 'autistic spectrum disorder':ti,ab 1,277

#193 'autism spectrum disorders':ti,ab 13,768

#192 'autism spectrum disorder':ti,ab 24,586

#191 'autism'/exp 82,206

#190 'neonates':ti,ab 101,672

#189 'neonate':ti,ab 38,625

#188 'newborns':ti,ab 78,314

#187 'newborn infants':ti,ab 20,432

#186 'newborn infant':ti,ab 8,618

#185 'infants, newborn':ti,ab 57

#184 'infant, newborn':ti,ab 107

#183 'newborn'/exp 628,687

#182 'pregnancy, multiple':ti,ab 367

#181 'pregnancies, multiple':ti,ab 69

#180 'multiple pregnancies':ti,ab 4,864

#179 'multiple pregnancy'/exp 27,754

#178 'wellbeing, child':ti,ab 10

#177 'child wellbeing':ti,ab 468

#176 'well-being, child':ti,ab 25

#175 'child well-being':ti,ab 563

#174 'well being, child':ti,ab 25

#173 'child well being':ti,ab 563

#172 'health, childrens':ti,ab 1

#171 'childrens health':ti,ab 80

#170 'health, child':ti,ab 292

#169 'neonatal deaths':ti,ab 4,806

#168 'deaths, neonatal':ti,ab 59

#167 'death, neonatal':ti,ab 304

#166 'neonatal death':ti,ab 7,205

#165 'perinatal deaths':ti,ab 2,524

#164 'deaths, perinatal':ti,ab 49

#163 'death, perinatal':ti,ab 82

#162 'perinatal death'/exp 4,287

#161 'health of the newborn infant':ti,ab 9

#160 'infant health, newborn':ti,ab 0

#159 'health, newborn infant':ti,ab 0

#158 'newborn infant health':ti,ab 2

#157 'health of newborn infants':ti,ab 24

#156 'health, neonatal':ti,ab 35

#155 'neonatal health':ti,ab 1,686

#154 'health, newborn':ti,ab 41

#153 'newborn health':ti,ab 1,472

#152 'health, baby':ti,ab 14

#151 'baby health':ti,ab 72

#150 'infant health':ti,ab 3,401

#149 'health, infant':ti,ab 84

#148 'child health'/exp 30,308

#147 'fetal growth restriction':ti,ab 7,504

#146 'intrauterine growth restriction':ti,ab 9,564

#145 'growth retardation, intrauterine':ti,ab 36

#144 'intrauterine growth retardation':ti,ab 7,301

#143 'fetal growth retardation':ti,ab 1,836

#142 'multigravidities':ti,ab 0

#141 'multigravidity':ti,ab 60

#140 'primigravidities':ti,ab 0

#139 'primigravidity':ti,ab 85

#138 'nulligravidities':ti,ab 0

#137 'nulligravidity':ti,ab 41

#136 'gravidities':ti,ab 72

#135 'gravidity':ti,ab 3,978

#134 'pregnancy'/exp 809,987

#133 'women, pregnant':ti,ab 893

#132 'woman, pregnant':ti,ab 116

#131 'pregnant women':ti,ab 138,627

#130 'pregnant woman'/exp 92,003

#129 'intrauterine growth retardation'/exp 48,388

#128 'neonatal complication'/exp 20

#127 'maternal complication'/exp 24

#126 'large for gestational age'/exp 3,751

#125 'operative vaginal delivery'/exp 44

#124 'small for date infant'/exp 17,250

#123 'low birth weights':ti,ab 635

#122 'birth weights, low':ti,ab 8

#121 'birth weight, low':ti,ab 321

#120 'low birth weight':ti,ab 37,534

#119 'low-birth-weight infants':ti,ab 9,508

#118 'low birth weight infant':ti,ab 1,240

#117 'infants, low-birth-weight':ti,ab 42

#116 'infant, low-birth-weight':ti,ab 79

#115 'low-birth-weight infant':ti,ab 1,240

#114 'low birth weight'/exp 66,723

#113 'preterm births':ti,ab 4,376

#112 'births, preterm':ti,ab 88

#111 'birth, preterm':ti,ab 397

#110 'preterm birth':ti,ab 27,704

#109 'premature births':ti,ab 1,275

#108 'births, premature':ti,ab 29

#107 'birth, premature':ti,ab 120

#106 'premature birth':ti,ab 5,134

#105 'prematurity'/exp 120,461

#104 'postcesarean section':ti,ab 76

#103 'c-sections (ob)':ti,ab 1

#102 'c section (ob)':ti,ab 0

#101 'c-section (ob)':ti,ab 0

#100 'abdominal delivery':ti,ab 498

#99 'caesarean sections':ti,ab 4,668

#98 'caesarean section':ti,ab 29,004

#97 'deliveries, abdominal':ti,ab 7

#96 'abdominal deliveries':ti,ab 105

#95 'delivery, abdominal':ti,ab 37

#94 'cesarean sections':ti,ab 4,833

#93 'cesarean section'/exp 111,631

#92 'proteinuria edema hypertension gestosis':ti,ab 0

#91 'gestosis, proteinuria-edema-hypertension':ti,ab 0

#90 'proteinuria-edema-hypertension gestosis':ti,ab 0

#89 'preeclampsia eclampsia 1s':ti,ab 0

#88 'eclampsia 1s, preeclampsia':ti,ab 0

#87 'eclampsia 1, preeclampsia':ti,ab 0

#86 '1s, preeclampsia eclampsia':ti,ab 0

#85 '1, preeclampsia eclampsia':ti,ab 3

#84 'preeclampsia eclampsia 1':ti,ab 7

#83 'toxemias, pregnancy':ti,ab 0

#82 'gestosis, eph':ti,ab 10

#81 'eph gestosis':ti,ab 531

#80 'toxemias, eph':ti,ab 3

#79 'toxemia, eph':ti,ab 2

#78 'eph toxemia':ti,ab 6

#77 'eph toxemias':ti,ab 0

#76 'eph complex':ti,ab 7

#75 'toxemia of pregnancies':ti,ab 1

#74 'pregnancy, toxemia of':ti,ab 14

#73 'pregnancies, toxemia of':ti,ab 1

#72 'of pregnancy, toxemia':ti,ab 155

#71 'of pregnancies, toxemia':ti,ab 0

#70 'toxemia of pregnancy':ti,ab 1,110

#69 'hypertension edema proteinuria gestosis':ti,ab 0

#68 'gestosis, hypertension-edema-proteinuria':ti,ab 0

#67 'hypertension-edema-proteinuria gestosis':ti,ab 0

#66 'gestosis, edema-proteinuria-hypertension':ti,ab 3

#65 'edema proteinuria hypertension gestosis':ti,ab 12

#64 'gestosis':ti,ab 1,524

#63 'edema-proteinuria-hypertension':ti,ab 51

#62 'toxemia, pregnancy':ti,ab 9

#61 'pregnancy toxemia':ti,ab 711

#60 'pregnancy toxemias':ti,ab 570

#59 'pre eclampsia':ti,ab 16,312

#58 'pre-eclampsia':ti,ab 16,312

#57 'preeclampsia'/exp 65,908

#56 'pregnancy transient hypertension':ti,ab 5

#55 'hypertension, pregnancy transient':ti,ab 0

#54 'transient hypertension, pregnancy':ti,ab 0

#53 'hypertension, gestational':ti,ab 466

#52 'gestational hypertension':ti,ab 5,205

#51 'induced hypertensions, pregnancy':ti,ab 0

#50 'induced hypertension, pregnancy':ti,ab 6

#49 'hypertensions, pregnancy induced':ti,ab 0

#48 'pregnancy induced hypertension':ti,ab 5,434

#47 'pregnancy-induced hypertension':ti,ab 5,434

#46 'hypertension, pregnancy induced':ti,ab 52

#45 'maternal hypertension'/exp 25,831

#44 'gestational diabetes mellitus':ti,ab 13,770

#43 'diabetes mellitus, gestational':ti,ab 106

#42 'gestational diabetes':ti,ab 26,984

#41 'pregnancy-induced diabetes':ti,ab 16

#40 'diabetes, pregnancy induced':ti,ab 62

#39 'diabetes, pregnancy-induced':ti,ab 62

#38 'pregnancy diabetes mellitus'/exp 41,740

#37 'complications, labor':ti,ab 23

#36 'labor complication':ti,ab 28

#35 'complication, labor':ti,ab 3

#34 'labor complications':ti,ab 257

#33 'obstetric labor complication':ti,ab 0

#32 'labor complications, obstetric':ti,ab 0

#31 'labor complication, obstetric':ti,ab 0

#30 'complications, obstetric labor':ti,ab 0

#29 'complication, obstetric labor':ti,ab 0

#28 'labor complication'/exp 222,620

#27 'complications, pregnancy':ti,ab 210

#26 'pregnancy complication':ti,ab 1,204

#25 'complication, pregnancy':ti,ab 18

#24 'pregnancy complication'/exp 164,704

#23 'obstetric labor':ti,ab 44

#22 'labor'/exp 46,035

#21 'obstetric delivery':ti,ab 169

#20 'obstetric deliveries':ti,ab 73

#19 'deliveries, obstetric':ti,ab 19

#18 'obstetric delivery'/exp 201,630

#17 #1 OR #2 OR #3 OR #4 OR #5 OR #6 OR #7 OR #8 OR #9 OR #10 OR #11 OR #12 OR #13 OR #14 OR #15 OR #16

#16 'sclerocystic ovary':ti,ad 41

#15 'ovary, sclerocystic':ti,ad 0

#14 'sclerocystic ovaries':ti,ad 34

#13 'polycystic ovary syndrome 1':ti,ad 11

#12 'ovarian syndrome, polycystic':ti,ad 0

#11 'polycystic ovarian syndrome':ti,ad 2,257

#10 'sclerocystic ovary syndrome':ti,ad 33

#9 'ovarian degeneration, sclerocystic':ti,ad 0

#8 'sclerocystic ovarian degeneration':ti,ad 2

#7 'syndrome, stein-leventhal':ti,ad 8

#6 'stein leventhal syndrome':ti,ad 525

#5 'stein-leventhal syndrome':ti,ad 525

#4 'polycystic ovary syndrome':ti,ad 12,711

#3 'syndrome, polycystic ovary':ti,ad 8

#2 'ovary syndrome, polycystic':ti,ad 3

#1 'ovary polycystic disease'/exp

Database: Cochrane library:

Search Strategy:

#1 MeSH descriptor: [Polycystic Ovary Syndrome] explode all trees 1614

#2 MeSH descriptor: [Delivery, Obstetric] explode all trees 5473

#3 MeSH descriptor: [Labor, Obstetric] explode all trees 2425

#4 MeSH descriptor: [Pregnancy Complications] explode all trees 12489

#5 MeSH descriptor: [Obstetric Labor Complications] explode all trees 4145

#6 MeSH descriptor: [Diabetes, Gestational] explode all trees 1053

#7 MeSH descriptor: [Hypertension, Pregnancy-Induced] explode all trees 1216

#8 MeSH descriptor: [Pre-Eclampsia] explode all trees 1033

#9 MeSH descriptor: [Cesarean Section] explode all trees 3282

#10 MeSH descriptor: [Premature Birth] explode all trees 1617

#11 MeSH descriptor: [Infant, Low Birth Weight] explode all trees 2269

#12 MeSH descriptor: [Infant, Small for Gestational Age] explode all trees 289

#13 MeSH descriptor: [Pregnant Women] explode all trees 377

#14 MeSH descriptor: [Gravidity] explode all trees 62

#15 MeSH descriptor: [Fetal Growth Retardation] explode all trees 410

#16 MeSH descriptor: [Infant Health] explode all trees 57

#17 MeSH descriptor: [Perinatal Death] explode all trees 87

#18 MeSH descriptor: [Child Health] explode all trees 132

#19 MeSH descriptor: [Pregnancy, Multiple] explode all trees 264

#20 MeSH descriptor: [Infant, Newborn] explode all trees 16781

#21 MeSH descriptor: [Autism Spectrum Disorder] explode all trees 1675

#22 #2 OR #3 OR #4 OR #5 OR #6 OR #7 OR #8 OR #9 OR #10 OR #11 OR #12 OR #13 OR #14 OR #15 OR #16 OR #17 OR #18 OR #19 OR #20 OR #21 32519

#23 #1 AND #22 84

Supplemental Table 2. Characters of included studies

| **Article No.** | **Author** | **Year** | **Country** | **Race** | **Study Design** | **No. of PCOS/control** | **Control Group** | **Outcomes** | **Type of**  **embryos**  **transferred** | **Adjusted confounders** | **Age (years)** | | **BMI(kg/m2)** | |
| --- | --- | --- | --- | --- | --- | --- | --- | --- | --- | --- | --- | --- | --- | --- |
|  |  |  |  |  |  |  |  |  |  |  | **PCOS** | **Control** | **PCOS** | **Control** |
| 1 | Urman et al. | 1992 | Canada | Caucasian | Retrospective | 4/10 | Patients with tubal factor infertility | Abortion | Unknown | Age | NA | NA | NA | NA |
| 2 | Homburg et al. | 1993 | Israel | Mediterranean | Retrospective | 47/38 | Patients with tubal factor infertility | Abortion | Unknown | Age | 32.4 ± 4.5 | 32.4 ± 2.8 | NA | NA |
| 3 | Hardy et al. | 1995 | Britain | Caucasian | Prospective | 5/4 | Patients with tubal factor infertility | Abortion | Unknown | Not controlled | NA | NA | NA | NA |
| 4 | Kodama et al. | 1995 | Japan | East Asian | Retrospective | 14/126 | Patients with tubal factor infertility and Unexplained infertility | Abortion | Unknown | Not controlled | 30.5 ± 2.1 | 31.0 ± 2.2 | NA | NA |
| 5 | Sengoku et al. | 1997 | Japan | East Asian | Retrospective | 4/6 | Patients with tubal factor infertility | Abortion | Unknown | Age | 32.5 ± 2.4 | 32.0 ± 3.0 | NA | NA |
| 6 | Doldi et al. | 1999 | Italy | Mediterranean | Retrospective | 19/16 | Patients with tubal factor infertility | Abortion | Unknown | Not controlled | 32.7 ± 0.7 | 33.4 ± 0.4 | 23.5 ± 0.9 | 21.2 ± 0.8 |
| 7 | Lesser et al. | 1997 | America | Caucasian | Retrospective | 24/44 | Non-PCOS | GDM | Unknown | Not controlled | 29.8 ± 5.3 | 32.0 ± 4.6 | 28.4 ± 4.7 | 23.4 ± 2.79 |
| 8 | FridstrÖM et al. | 1999 | Sweden | Caucasian | Retrospective | 33/66 | Non-PCOS | Birth weight, preterm | Unknown | Birth weights adjusted for gestational age at delivery | 32 ± 4 | 33 ± 3.6 | 24.5 ± 4.2 | 23.2 ± 3.4 |
| 9 | Bjercke et al. | 2002 | Norway | Caucasian | Retrospective | 29/335 | Non-PCOS | GDM, PIH, PE, preterm, birthweight, NICU admission | Unknown | IR | 31.5 ± 3.8 | 32.7 ± 3.4 | 25.2 ± 3.9 | 21.9 ± 2.7 |
| 10 | Mulders et al. | 2003 | Netherlands | Caucasian | Retrospective | 16/12 | Patients with tubal factor infertility | Abortion | Unknown | Age | 34.9 ± 3.2 | 34.8 ± 3.5 | 26.5 ± 6.2 | 25.0 ± 5.2 |
| 11 | Urman et al. | 2004 | America | Caucasian | Retrospective | 91/81 | Non-PCOS | Abortion | Unknown | Age, duration of infertility | 31.83 | 31.27 | NA | NA |
| 12 | Kuivasaari-Pirinen et al. | 2009 | Finland | Caucasian | Retrospective | 66/106 | Patients with tubal factor infertility | Abortion | Fresh and frozen ET | Not controlled | 29.8 ± 4.3 | 33.1 ± 4.6 | 27.3 ± 6.8 | 24.5 ± 4.9 |
| 13 | Han et al. | 2011 | Korea | East Asian | Retrospective | 336/1003 | Patients with tubal factor infertility | GDM, PIH, preterm, abortion, SGA, LGA | Fresh and frozen ET | Not controlled | 31.6 ± 3.1  31.2 ± 2.7 | 32.2 ± 3.2  32.5 ± 2.8 | 27.46 ± 2.42  20.45 ± 2.02 | 27.50 ± 2.00  20.58 ± 1.97 |
| 14 | Nejad et al. | 2011 | Iran | Caucasian | Prospective | 183/183 | Patients with tubal factor infertility | Abortion | Unknown | Not controlled | 29.5 ± 0.3 | 30.09 ± 0.3 | NA | NA |
| 15 | Li et al. | 2013 | China | East Asian | Retrospective | 104/576 | Non-PCOS | Abortion | Fresh ET | Age | 33(30-36) | 33(31-35) | 22.2 (20.7–24.9) | 21.2 (19.6–22.7) |
| 16 | Liu et al. | 2014 | China | East Asian | Retrospective | 301/3591 | Non-PCOS | Abortion | Fresh and frozen ET separately | Number and stage of ET | 31.7 ± 5.2  30.5 ± 3.5  30.8 ± 3.6  30.0 ± 3.5 | 31.9 ± 4.3  31.6 ± 4.3  30.9 ± 3.9  30.6 ± 3.9 | NA | NA |
| 17 | Wan et al. | 2015 | China | East Asian | Retrospective | 24/171 | Non-PCOS | GDM, PIH, PE, preterm, Congenital malformations, birth weight, NICU admission | Unknown | Age and multiple pregnancy | 31.4 ± 2 | 32.7 ± 3.1 | 22.8 ± 3.6 | 21.5 ± 2.6 |
| 18 | Sterling et al. | 2016 | Canada | Caucasian | Retrospective | 173/911 | Non-PCOS | GDM, PIH, preterm, LGA | Fresh ET | Age, parity, BMI and time to conception | 33(30 - 35) | 35 (32-37) | 22.7(20.4–28.3) | 22.6 (20.8–26.0) |
| 19 | Chen et al. | 2017 | China | East Asian | Prospective | 59/120 | Patients with tubal factor infertility or male factor infertility | Abortion | Fresh ET | Not controlled | 29.1 ± 3.5 | 30.3 ± 3.9 | 22.2 ± 3.2 | 21.4 ± 2.9 |
| 20 | Su Liu et al. | 2020 | China | East Asian | Retrospective | 666/7012 | Non-PCOS | GDM, PIH, abortion | Unknown | Not controlled | 30.0 (27.0–32.0) | 31.0 (29.0–34.0) | 22.3 (20.3–25.0) | 20.7 (19.2–22.6) |
| 21 | Valdimarsdottir et al. | 2021 | Sweden | Caucasian | Retrospective | 159/320 | Non-PCOS | GDM, PIH, PE, preterm, birth weight, LGA | Unknown | Not controlled | 31.7 ± 4.6 | 30.2 ± 5.1 | 26.2 ± 5.8 | 26.1 ± 5.4 |
| 22 | Hu et al. | 2021 | China | East Asian | Retrospective | 338/1783 | Non-PCOS | GDM, PIH, preterm, abortion, Placenta praevia, Congenital malformations, birth weight | Frozen ET | Not controlled | 29.75 ± 3.64 | 31.05 ± 3.95 | 22.56 ± 3.27 | 21.51 ± 2.77 |
| 23 | Abdulkhalikova et al. | 2021 | Slovenia | Caucasian | Retrospective | 73/196 | Non-PCOS | GDM, PIH, PE, preterm, birth weight, NICU admission, SGA | Fresh ET | Not controlled | 33.5 ± 3.9  33.4 ± 4.3 | 34.6 ± 3.9  33.6 ± 4.0 | NA | NA |
| 24 | Lin et al. | 2021 | China | East Asian | Retrospective | 1167/9995 | Patients with tubal factor infertility or male factor infertiliy | Preterm, birth weight | Frozen ET | Not controlled | NA | NA | NA | NA |
| 25 | Yurci et al. | 2021 | Turkey | Mediterranean | Retrospective | 178/259 | Non-PCOS | Congenital malformations | Unknown | Not controlled | NA | NA | NA | NA |
| 26 | Cai et al. | 2021 | China | East Asian | Retrospective | 2357/19463 | Non-PCOS | Abortion | Fresh and frozen ET | Not controlled | 29.0 ± 3.3 | 30.5 ± 4.2 | 24.3 ± 3.7 | 22.3 ± 3.2 |
| 27 | Jie et al. | 2022 | China | East Asian | Retrospective | 336/2325 | Patients who underwent IVF/ICSI due to fallopian tubal factor or male factor infertility | preterm, abortion, birth weight | Frozen ET | Not controlled | 30, 5 | 30, 7 | 21.6, 3.8 | 21.6, 4.1 |
| 28 | Wang, Zheng et al. | 2022 | China | East Asian | Retrospective | 1253/4416 | Non-PCOS | preterm, abortion | Frozen ET | Not controlled | NA | NA | NA | NA |
| 29 | Qiu et al. | 2022 | China | East Asian | Retrospective | 1876/14630 | Patients with tubal factor infertility or male factor infertiliy | GDM, PIH, PE, preterm, PROM, macrosomia, SGA, LGA, Congenital malformations | Frozen ET | Not controlled | NA | NA | NA | NA |
| 30 | Shinde et al. | 2022 | India | Indian | Prospective | 54/54 | Non-PCOS | Preterm, SGA, LGA, NICU admission | Frozen ET | Not controlled | NA | NA | NA | NA |
| 31 | Jiang et al. | 2022 | China | East Asian | Retrospective | 30/31 | Patients with tubal factor infertility | GDM, PIH, pretem, abortion | Unknown | Not controlled | 32.41 ± 4.55 | 33.66 ± 3.82 | 25.10 ± 4.15 | 23.03 ± 3.69 |
| 32 | Ni et al. | 2022 | China | East Asian | Retrospective | 1384/14606 | Patients with tubal factor infertility or male factor infertiliy | Preterm, abortion, Congenital malformations, SGA, LGA | Frozen ET | Not controlled | 30.58 ± 3.49 | 30.63 ± 3.60 | 23.58 ± 6.08 | 21.53 ± 5.03 |
| 33 | Wang et al. | 2022 | China | East Asian | Retrospective | 346/2265 | Non-PCOS | GDM, PIH, preterm, abortion | Fresh ET | Not controlled | NA | NA | NA | NA |

Supplemental Table 3. Assessment of risk of bias based on Newcastle-Ottawa Scale.

| **Author** | | **Selection** | **Conparability**^a^ | **Exposure / Outcome** | **Total** |
| --- | --- | --- | --- | --- | --- |
| Urman et al. | 1992 | *** | ** | ** | 7 |
| Homburg et al. | 1993 | *** | ** | ** | 7 |
| Hardy et al. | 1995 | **** | ** | ** | 8 |
| Kodama et al. | 1995 | *** | ** | ** | 7 |
| Sengoku et al. | 1997 | *** | ** | ** | 7 |
| Doldi et al. | 1999 | *** | ** | ** | 7 |
| Mulders et al. | 2003 | *** | ** | ** | 7 |
| Urman et al. | 2004 | ** | ** | ** | 6 |
| Lesser et al. | 1997 | ** | ** | *** | 7 |
| FridstrÖM et al. | 1999 | ** | ** | ** | 6 |
| Bjercke et al. | 2002 | ** | ** | *** | 7 |
| Kuivasaari-Pirinen et al. | 2009 | *** | ** | ** | 7 |
| Han et al. | 2011 | *** | ** | *** | 8 |
| Nejad et al. | 2011 | **** | ** | ** | 9 |
| Li et al. | 2013 | ** | ** | ** | 6 |
| Liu et al. | 2014 | ** | ** | *** | 7 |
| Wan et al. | 2015 | ** | ** | *** | 7 |
| Sterling et al. | 2016 | ** | ** | ** | 6 |
| Chen et al. | 2017 | **** | ** | ** | 8 |
| Su Liu et al. | 2020 | ** | ** | ** | 6 |
| Valdimarsdottir et al. | 2021 | *** | ** | *** | 8 |
| Hu et al. | 2021 | ** | ** | *** | 7 |
| Abdulkhalikova et al. | 2021 | ** | ** | *** | 7 |
| Lin et al. | 2021 | *** | ** | ** | 7 |
| Yurci et al. | 2021 | ** | ** | *** | 7 |
| Cai et al. | 2021 | ** | ** | *** | 7 |
| Jie et al. | 2022 | *** | ** | *** | 8 |
| Wang, Zheng et al. | 2022 | ** | ** | *** | 7 |
| Qiu et al. | 2022 | *** | ** | *** | 8 |
| Shinde et al. | 2022 | *** | ** | *** | 8 |
| Jiang et al. | 2022 | *** | ** | ** | 7 |
| Ni et al. | 2022 | *** | ** | *** | 8 |
| Wang et al. | 2022 | ** | ** | ** | 6 |

^a^ Except for Comparability which can be awarded a maximum of two stars, other items were given a maximum of one star for each study.

Supplemental Table 4. Subgroups with pregrancy complications and neonatal outcomes

| **Outcomes** | **Subgroup analysis** | | | | | |
| --- | --- | --- | --- | --- | --- | --- |
|  | **Ethnicity** | **Transfer method** | **BMI category** | **Study design** | **NOS quality** | **With or without hyperandrogenism** |
| GDM | Caucasian, East Asian, Indian | Fresh ET, Frozen ET | Overweight/obesity, Normal weight/lean | Retrospective, prospective | High,  Middle | NA |
| PIH | Caucasian, East Asian | Fresh ET, Frozen ET | Overweight/obesity, Normal weight/lean | Retrospective * | High,  Middle | NA |
| PE | Caucasian, East Asian | Fresh ET, Frozen ET | NA | Retrospective, prospective | High,  Middle | Yes,  No |
| Preterm birth | Caucasian, East Asian, Indian | Fresh ET, Frozen ET | Overweight/obesity, Normal weight/lean | Retrospective, prospective | High,  Middle | Yes,  No |
| Abortion | Caucasian, East Asian | Fresh ET, Frozen ET | NA | Retrospective, prospective | High,  Middle | NA |
| CA | Caucasian, East Asian, Mediterranean | Fresh ET, Frozen ET | NA | Retrospective * | High,  Middle | NA |
| SGA | Caucasian, East Asian, Indian | Fresh ET, Frozen ET | Overweight/obesity, Normal weight/lean | Retrospective, prospective | High,  Middle | NA |
| LGA | Caucasian, East Asian, Indian | Fresh ET, Frozen ET | NA | Retrospective, prospective | High,  Middle | NA |
| LBW | Caucasian, East Asian, Indian | Fresh ET, Frozen ET | NA | Retrospective, prospective | High,  Middle | NA |
| Macrosomia | Caucasian, East Asian | Fresh ET, Frozen ET | NA | Retrospective * | High,  Middle | NA |
| NICU admission | Caucasian, East Asian, Indian | Fresh ET, Frozen ET | NA | Retrospective, prospective | High * | NA |
| Birth weight | Caucasian, East Asian | Fresh ET, Frozen ET | NA | Retrospective * | High,  Middle | NA |

* means all eligible studies were classified in this subgroup

Supplemental Table 5. Subgroup analyses for GDM.

|  | **No. of studies** | **OR (95% CI)** | ***P*** | **Weight(%)** | ***Ph*** | ***I²* (%)** |
| --- | --- | --- | --- | --- | --- | --- |
| **All studies** | 15 | 1.51 (1.17, 1.94) | 0.001 | 100 | 0.01 | 51 |
| **Ethnicity** |  |  |  |  |  |  |
| Caucasian | 6 | 2.40 (1.39, 4.14) | 0.002 | 21.9 | 0.27 | 22 |
| East Asian | 8 | 1.25 (0.94, 1.67) | 0.13 | 72.4 | 0.01 | 61 |
| Indian | 1 | 2.80 (1.16, 6.74) | 0.02 | 5.7 |  |  |
| **Transfer method** |  |  |  |  |  |  |
| Frozen ET | 3 | 1.56 (0.90, 2.72) | 0.12 | 57 | 0.009 | 79 |
| Fresh ET | 4 | 1.80 (1.23, 2.62) | 0.002 | 43 | 0.33 | 13 |
| **BMI category** |  |  |  |  |  |  |
| Overweight/obesity | 2 | 1.31 (0.67, 2.59) | 0.43 | 53.2 | 1.00 | 0 |
| Normal weight/lean | 2 | 0.93 (0.41, 2.07) | 0.85 | 46.8 | 0.32 | 0 |
| **Study design** |  |  |  |  |  |  |
| Retrospective | 14 | 1.45 (1.12, 1.88) | 0.005 | 94.3 | 0.01 | 52 |
| prospective | 1 | 2.8 (1.16, 6.74) | 0.02 | 5.7 |  |  |
| **NOS quality** |  |  |  |  |  |  |
| High | 12 | 1.47 (1.07, 2.02) | 0.02 | 67.6 | 0.05 | 45 |
| Middle | 3 | 1.66 (0.96, 2.87) | 0.07 | 32.4 | 0.02 | 73 |

Supplemental Table 6. Subgroup analyses for PIH.

|  | **No. of studies** | **OR (95% CI)** | ***P*** | **Weight(%)** | ***Ph*** | ***I²* (%)** |
| --- | --- | --- | --- | --- | --- | --- |
| **All studies** | 15 | 1.72 (1.25, 2.39) | 0.001 | 100 | 0.004 | 57 |
| **Ethnicity** |  |  |  |  |  |  |
| Caucasian | 7 | 2.56 (1.14, 5.71) | 0.02 | 32.6 | 0.01 | 63 |
| East Asian | 8 | 1.53 (1.10, 2.12) | 0.01 | 67.4 | 0.04 | 53 |
| **Transfer method** |  |  |  |  |  |  |
| Frozen ET | 2 | 1.79 (1.03, 3.10) | 0.04 | 55.5 | 0.04 | 75 |
| Fresh ET | 4 | 1.86 (1.08, 3.21) | 0.03 | 44.5 | 0.19 | 37 |
| **BMI category** |  |  |  |  |  |  |
| Overweight/obesity | 2 | 0.55 (0.57, 6.56) | 0.63 | 23.2 | 0.10 | 64 |
| Normal weight/lean | 2 | 1.00 (0.56, 1.78) | 0.99 | 76.8 | 0.64 | 0 |
| **NOS quality** |  |  |  |  |  |  |
| High | 11 | 1.47 (0.92, 2.36) | 0.11 | 64.1 | 0.002 | 63 |
| Middle | 4 | 2.16 (1.41, 3.30) | 0.0004 | 35.9 | 0.20 | 36 |

Supplemental Table 7. Subgroup analyses for PE.

|  | **No. of studies** | **OR (95% CI)** | ***P*** | **Weight(%)** | ***Ph*** | ***I²* (%)** |
| --- | --- | --- | --- | --- | --- | --- |
| **All studies** | 8 | 2.12 (1.49, 3.02) | <0.0001 | 100 | 0.45 | 0 |
| **Ethnicity** |  |  |  |  |  |  |
| Caucasian | 5 | 2.92 (1.52, 5.62) | 0.001 | 25.9 | 0.46 | 0 |
| East Asian | 2 | 1.51 (0.91, 2.50) | 0.11 | 57.7 | 0.66 | 0 |
| Indian | 1 | 3.02 (1.26, 7.26) | 0.01 | 16.3 |  |  |
| **Transfer method** |  |  |  |  |  |  |
| Frozen ET | 2 | 1.82 (1.17, 2.83) | 0.008 | 93.1 | 0.16 | 49 |
| Fresh ET | 2 | 2.65 (0.56, 12.51) | 0.22 | 6.9 | 0.77 | 0 |
| **Study design** |  |  |  |  |  |  |
| Retrospective | 7 | 1.94 (1.31, 2.87) | 0.0009 | 83.7 | 0.44 | 0 |
| prospective | 1 | 3.02 (1.26, 7.26) | 0.01 | 16.3 |  |  |
| **NOS quality** |  |  |  |  |  |  |
| High | 7 | 2.01 (1.40, 2.88) | 0.0001 | 99.2 | 0.54 | 0 |
| Middle | 1 | 15.26 (0.76, 304.73) | 0.07 | 0.8 |  |  |

Supplemental Table 8. Subgroup analyses for preterm birth.

|  | **No. of studies** | **OR (95% CI)** | ***P*** | **Weight(%)** | ***Ph*** | ***I²* (%)** |
| --- | --- | --- | --- | --- | --- | --- |
| **All studies** | 18 | 1.29 (1.21, 1.39) | <0.00001 | 100 | 0.31 | 12 |
| **Ethnicity** |  |  |  |  |  |  |
| Caucasian | 5 | 1.32 (0.86, 2.02) | 0.21 | 2.7 | 0.49 | 0 |
| East Asian | 12 | 1.29 (1.20, 1.39) | <0.00001 | 96.9 | 0.19 | 26 |
| Indian | 1 | 2.23 (0.71, 7.02) | 0.17 | 0.3 |  |  |
| **Transfer method** |  |  |  |  |  |  |
| Frozen ET | 7 | 1.31 (1.21, 1.42) | <0.00001 | 91.8 | 0.07 | 48 |
| Fresh ET | 3 | 1.30 (0.99, 1.71) | 0.06 | 8.2 | 0.30 | 18 |
| **BMI category** |  |  |  |  |  |  |
| Overweight/obesity | 2 | 0.66 (0.36, 1.21) | 0.18 | 25.4 | 0.70 | 0 |
| Normal weight/lean | 2 | 1.24 (0.92, 1.66) | 0.15 | 74.6 | 0.63 | 0 |
| **Study design** |  |  |  |  |  |  |
| Retrospective | 17 | 1.29 (1.20, 1.39) | <0.00001 | 99.7 | 0.30 | 13 |
| prospective | 1 | 2.23 (0.71, 7.02) | 0.17 | 0.3 |  |  |
| **NOS quality** |  |  |  |  |  |  |
| High | 15 | 1.29 (1.19, 1.39) | <0.00001 | 85.7 | 0.18 | 25 |
| Middle | 3 | 1.33 (1.11, 1.60) | 0.003 | 14.3 | 0.74 | 0 |
| **With or without hyperandrogenism** |  |  |  |  |  |  |
| With hyperandrogenism | 3 | 1.69 (1.31, 2.18) | <0.0001 | 31.2 | 0.45 | 0 |
| Without hyperandrogenism | 3 | 1.24 (1.02, 1.50) | 0.03 | 68.8 | 0.71 | 0 |

Supplemental Table 9. Subgroup analyses for abortion.

|  | **No. of studies** | **OR (95% CI)** | ***P*** | **Weight(%)** | ***Ph*** | ***I²* (%)** |
| --- | --- | --- | --- | --- | --- | --- |
| **All studies** | 26 | 0.97 (0.82, 1.15) | 0.73 | 100 | <0.0001 | 63 |
| **Ethnicity** |  |  |  |  |  |  |
| Caucasian | 6 | 1.37 (0.80, 2.34) | 0.25 | 8.1 | 0.64 | 0 |
| East Asian | 18 | 0.95 (0.79, 1.14) | 0.58 | 88.4 | <0.00001 | 72 |
| Mediterranean | 2 | 0.78 (0.26, 2.33) | 0.66 | 3.5 | 0.22 | 35 |
| **Transfer method** |  |  |  |  |  |  |
| Frozen ET | 6 | 1.00 (0.78, 1.28) | 0.99 | 72.1 | 0.001 | 75 |
| Fresh ET | 5 | 1.41 (1.08, 1.84) | 0.01 | 27.9 | 0.40 | 1 |
| **Study design** |  |  |  |  |  |  |
| Retrospective | 23 | 0.99 (0.83, 1.17) | 0.81 | 96.5 | <0.00001 | 66 |
| prospective | 3 | 0.63 (0.27, 1.44) | 0.27 | 3.5 | 0.95 | 0 |
| **NOS quality** |  |  |  |  |  |  |
| High | 22 | 0.98 (0.87, 1.11) | 0.78 | 79.8 | 0.09 | 31 |
| Middle | 4 | 1.10 (0.40, 3.02) | 0.86 | 20.2 | <0.00001 | 92 |
| **With or without hyperandrogenism** |  |  |  |  |  |  |
| With hyperandrogenism | 4 | 1.38 (1.12, 1.71) | 0.003 | 31.8 | 0.64 | 0 |
| Without hyperandrogenism | 3 | 1.23 (1.06, 1.43) | 0.007 | 68.2 | 0.24 | 29 |

Supplemental Table 10. Subgroup analyses for CA.

|  | **No. of studies** | **OR (95% CI)** | ***P*** | **Weight(%)** | ***Ph*** | ***I²* (%)** |
| --- | --- | --- | --- | --- | --- | --- |
| **All studies** | 6 | 1.28 (0.83, 1.97) | 0.26 | 100 | 0.06 | 54 |
| **Ethnicity** |  |  |  |  |  |  |
| Caucasian | 1 | 0.91 (0.19, 4.23) | 0.90 | 2.3 |  |  |
| East Asian | 4 | 0.99 (0.79, 1.25) | 0.96 | 93.4 | 0.26 | 26 |
| Mediterranean | 1 | 3.13 (1.37, 7.13) | 0.007 | 4.3 |  |  |
| **Transfer method** |  |  |  |  |  |  |
| Frozen ET | 3 | 0.98 (0.77, 1.24) | 0.85 | 97.6 | 0.20 | 37 |
| Fresh ET | 1 | 0.91 (0.19, 4.23) | 0.90 | 2.4 |  |  |
| **NOS quality** |  |  |  |  |  |  |
| High | 5 | 1.33 (0.83, 2.14) | 0.23 | 93.6 | 0.03 | 63 |
| Middle | 1 | 0.91 (0.19, 4.23) | 0.90 | 6.4 |  |  |

Supplemental Table 11. Subgroup analyses for SGA.

|  | **No. of studies** | **OR (95% CI)** | ***P*** | **Weight(%)** | ***Ph*** | ***I²* (%)** |
| --- | --- | --- | --- | --- | --- | --- |
| **All studies** | 8 | 1.19 (0.69, 2.06) | 0.53 | 100 | <0.00001 | 81 |
| **Ethnicity** |  |  |  |  |  |  |
| Caucasian | 3 | 0.57 (0.21, 1.57) | 0.28 | 23.8 | 0.29 | 20 |
| East Asian | 4 | 1.26 (0.69, 2.33) | 0.45 | 70.8 | <0.00001 | 89 |
| Indian | 1 | 15.14 (1.89, 121.19) | 0.01 | 5.4 |  |  |
| **Transfer method** |  |  |  |  |  |  |
| Frozen ET | 3 | 0.95 (0.56, 1.64) | 0.86 | 78.6 | 0.01 | 77 |
| Fresh ET | 3 | 0.57 (0.21, 1.57) | 0.28 | 24.1 | 0.29 | 20 |
| **BMI category** |  |  |  |  |  |  |
| Overweight/obesity | 2 | 3.30 (0.01, 1304.80) | 0.70 | 42.1 | 0.0008 | 91 |
| Normal weight/lean | 2 | 1.07 (0.20, 5.71) | 0.94 | 57.9 | 0.09 | 65 |
| **Study design** |  |  |  |  |  |  |
| Retrospective | 7 | 1.03 (0.61, 1.74) | 0.90 | 94.6 | <0.0001 | 80 |
| Prospective | 1 | 15.14 (1.89, 121.19) | 0.01 | 5.4 |  |  |
| **NOS quality** |  |  |  |  |  |  |
| High | 7 | 1.24 (0.67, 2.27) | 0.49 | 86.7 | <0.00001 | 84 |
| Middle | 1 | 0.99 (0.36, 2.69) | 0.98 | 13.3 |  |  |

Supplemental Table 12. Subgroup analyses for LGA.

|  | **No. of studies** | **OR (95% CI)** | ***P*** | **Weight(%)** | ***Ph*** | ***I²* (%)** |
| --- | --- | --- | --- | --- | --- | --- |
| **All studies** | 7 | 1.11 (0.81, 1.51) | 0.52 | 100 | 0.003 | 70 |
| **Ethnicity** |  |  |  |  |  |  |
| Caucasian | 2 | 1.37 (0.33, 5.71) | 0.67 | 20.1 | 0.02 | 83 |
| East Asian | 4 | 1.01 (0.76, 1.33) | 0.96 | 78.8 | 0.01 | 72 |
| Indian | 1 | 9.71 (0.51, 184.97) | 0.13 | 1.1 |  |  |
| **Transfer method** |  |  |  |  |  |  |
| Frozen ET | 3 | 1.03 (0.91, 1.17) | 0.67 | 91.8 | 0.27 | 23 |
| Fresh ET | 1 | 2.78 (1.27, 6.10) | 0.01 | 8.2 |  |  |
| **Study design** |  |  |  |  |  |  |
| Retrospective | 6 | 1.08 (0.80, 1.46) | 0.62 | 98.9 | <0.00001 | 80 |
| prospective | 1 | 9.71 (0.51, 184.97) | 0.13 | 1.1 |  |  |
| **NOS quality** |  |  |  |  |  |  |
| High | 6 | 0.99 (0.75, 1.31) | 0.95 | 89.1 | 0.003 | 72 |
| Middle | 1 | 2.78 (1.27, 6.10) | 0.01 | 10.9 |  |  |

Supplemental Table 13. Subgroup analyses for LBW.

|  | **No. of studies** | **OR (95% CI)** | ***P*** | **Weight(%)** | ***Ph*** | ***I²* (%)** |
| --- | --- | --- | --- | --- | --- | --- |
| **All studies** | 9 | 1.29 (1.14, 1.47) | <0.0001 | 100 | 0.11 | 39 |
| **Ethnicity** |  |  |  |  |  |  |
| Caucasian | 3 | 1.25 (0.73, 2.14) | 0.41 | 6.2 | 0.57 | 0 |
| East Asian | 5 | 1.27 (1.11, 1.45) | 0.0005 | 93.0 | 0.09 | 49 |
| Indian | 1 | 4.38 (1.34, 14.33) | 0.01 | 0.8 |  |  |
| **Transfer method** |  |  |  |  |  |  |
| Frozen ET | 5 | 1.28 (0.99, 0.67) | 0.06 | 86.3 | 0.03 | 63 |
| Fresh ET | 3 | 1.27 (0.74, 2.16) | 0.38 | 13.7 | 0.57 | 0 |
| **Study design** |  |  |  |  |  |  |
| Retrospective | 6 | 1.27 (1.11, 1.45) | 0.0004 | 99.2 | 0.25 | 23 |
| Prospective | 1 | 4.38 (1.34, 14.33) | 0.01 | 0.8 |  |  |
| **NOS quality** |  |  |  |  |  |  |
| High | 6 | 1.29 (1.13, 1.47) | 0.0002 | 97.0 | 0.07 | 46 |
| Middle | 1 | 1.42 (0.68, 2.94) | 0.35 | 3.0 |  |  |

Supplemental Table 14. Subgroup analyses for macrosomia.

|  | **No. of studies** | **OR (95% CI)** | ***P*** | **Weight(%)** | ***Ph*** | ***I²* (%)** |
| --- | --- | --- | --- | --- | --- | --- |
| **All studies** | 8 | 1.00 (0.79, 1.28) | 1.00 | 100 | 0.02 | 58 |
| **Ethnicity** |  |  |  |  |  |  |
| Caucasian | 3 | 8.62 (0.43, 171.96) | 0.16 | 7.3 | 0.004 | 82 |
| East Asian | 5 | 0.92 (0.81, 1.04) | 0.18 | 92.7 | 0.38 | 4 |
| **Transfer method** |  |  |  |  |  |  |
| Frozen ET | 4 | 0.92 (0.81, 1.04) | 0.19 | 92.6 | 0.36 | 6 |
| Fresh ET | 3 | 8.62 (0.43, 171.96) | 0.16 | 7.4 | 0.004 | 82 |
| **NOS quality** |  |  |  |  |  |  |
| High | 7 | 1.01 (0.77, 1.32) | 0.97 | 94.1 | 0.01 | 64 |
| Middle | 1 | 1.01 (0.40, 2.55) | 0.98 | 5.9 |  |  |

Supplemental Table 15. Subgroup analyses for NICU admission.

|  | **No. of studies** | **OR (95% CI)** | ***P*** | **Weight(%)** | ***Ph*** | ***I²* (%)** |
| --- | --- | --- | --- | --- | --- | --- |
| **All studies** | 5 | 1.41 (0.55, 3.66) | 0.48 | 100 | 0.02 | 66 |
| **Ethnicity** |  |  |  |  |  |  |
| Caucasian | 3 | 1.15 (0.38, 3.43) | 0.81 | 65.2 | 0.05 | 66 |
| East Asian | 1 | 0.58 (0.13, 2.66) | 0.49 | 17.6 |  |  |
| Indian | 1 | 8.24 (1.76, 38.61) | 0.007 | 17.2 |  |  |
| **Transfer method** |  |  |  |  |  |  |
| Frozen ET | 1 | 8.24 (1.76, 38.61) | 0.007 | 31.9 |  |  |
| Fresh ET | 2 | 0.68 (0.16, 2.96) | 0.61 | 68.1 | 0.12 | 58 |
| **Study design** |  |  |  |  |  |  |
| Retrospective | 4 | 1.02 (0.41, 2.55) | 0.97 | 82.8 | 0.07 | 58 |
| Prospective | 1 | 8.24 (1.76, 38.61) | 0.007 | 17.2 |  |  |

Supplemental Table 16. Subgroup analyses for birth weight.

|  | **No. of studies** | **Mean Difference**  **(95% CI)** | ***P*** | **Weight(%)** | ***Ph*** | ***I²* (%)** |
| --- | --- | --- | --- | --- | --- | --- |
| **All studies** | 8 | -10.13 (-36.78, 16.52) | 0.46 | 100 | 0.07 | 47 |
| **Ethnicity** |  |  |  |  |  |  |
| Caucasian | 5 | 13.05 (-85.74, 111.83) | 0.80 | 32.1 | 0.37 | 7 |
| East Asian | 3 | 9.51 (-78.37, 97.40) | 0.83 | 67.9 | 0.01 | 76 |
| **Transfer method** |  |  |  |  |  |  |
| Frozen ET | 2 | 12.81 (-86.84, 112.47) | 0.80 | 83.5 | 0.004 | 88 |
| Fresh ET | 2 | 3.65 (-428.76, 436.06) | 0.99 | 16.5 | 0.06 | 71 |
| **NOS quality** |  |  |  |  |  |  |
| High | 7 | 10.61 (-55.53, 76.74) | 0.75 | 96.1 | 0.04 | 55 |
| Middle | 1 | -17.00 (-313.06, 279.06) | 0.91 | 3.9 |  |  |

Supplemental Table 17. Sensitive analyses and publication bias for pregnancy complications and neonatal outcomes

| **Outcome** | **No. of Studies** | **Effects Model** | **Variations in Sensitivity Analyses**  **OR (95%CI)** | **Egger's Test for Publication Bias (*P*)** | **Variations in Trim and Filled Analyses**  **OR (95%CI)** |
| --- | --- | --- | --- | --- | --- |
| **Comparison in pregnancy complications** | | | | |  |
| GDM | 15 | random | NS | 0.694 | - |
| PIH | 15 | random | NS | 0.494 | - |
| PE | 8 | fixed | NS | 0.170 | - |
| Preterm birth | 18 | fixed | NS | 0.450 | - |
| Abortion | 26 | random | NS | 0.620 | - |
| **Comparison in neonatal outcomes** | | | | |  |
| Congenital malformations | 6 | random | NS | 0.301 | - |
| SGA | 8 | random | NS | 0.497 | - |
| LGA | 7 | random | NS | 0.302 | - |
| Low birth weight | 9 | fixed | NS | 0.525 | - |
| Macrosomia | 8 | random | NS | 0.103 | - |
| NICU Admission | 5 | random | NS | 0.543 | - |
| Birth weight | 8 | fixed | -0.02 (-0.07, 0.03) | 0.453 | - |

Supplementary Table 18. Information from published meta-analyses on pregnancy complications and neonatal outcomes in women with PCOS.

| Author | Year | sample size  PCOS/control | Risk (OR/RR 95%CI) | Conclusion | Strengths | Limitations |
| --- | --- | --- | --- | --- | --- | --- |
| C.M.Boomsma et al. | 2006 | 720/4505 | GDM (OR 2.94; 95% CI: 1.70-5.08);  PIH (OR 3.67; 95% CI: 1.98-6.81);  PE (OR 3.47; 95% CI: 1.95-6.17);  preterm birth (OR 1.75; 95% CI: 1.16-2.62);  admission to NICU (OR 2.31; 95% CI: 1.25-4.26);  perinatal mortality (OR 3.07; 95% CI: 1.03-9.21). | Women with PCOS are at increased risk of pregnancy and neonatal complications. | Known confounding variables (such as BMI) were minimized by performing subgroup analyses on higher validity studies. | Comparison of outcomes from multiple pregnancy in PCOS with controls was not possible because of lack of stratification in the studies analysed. |
| Lucinda E. Kjerulff et al. | 2011 | 2544/89848 | GDM (OR 2.82;95% CI, 1.93-4.10);  PIH (OR 4.07; 95% CI, 2.75-6.02);  PE (OR 4.23; 95% CI, 2.77-6.46);  preterm birth (OR 2.20; 95%CI, 1.59-3.04);  cesarean delivery (OR 1.41; 95% CI, 0.96-2.07);  operative vaginal delivery (OR 1.56; 95% CI, 0.93-2.63);  SGA (OR 2.62; 95% CI, 1.35-5.10);  LGA(OR 1.56; 95% CI, 0.92-2.64). | Higher association of pregnancy complications and PCOS compared with patients who do not have PCOS.  A stronger association between PCOS and hypertensive disorders than has been shown previously. | It confirms earlier findings regarding obstetrics patients with PCOS and also updates ORs for associated pregnancy complications. | Inherent to the nature of observational studies: heterogeneity and lack of prospective data to establish causation. |
| Jun Z Qin et al. | 2013 | 4982/119692 | GDM (OR 3.43; 95% CI: 2.49-4.74);  PIH (OR 3.43;95% CI: 2.49-4.74);  preeclampsia (OR 2.17; 95% CI: 1.91-2.46);  preterm birth (OR 1.93; 95%CI: 1.45-2.57);  caesarean section (OR 1.74; 95% CI: 1.38-2.11);  birth weight (WMD -0.11g; 95%CI: -0.19 - -0.03);  admission to NICU (OR 2.32; 95% CI: 1.40-3.85). | Women with PCOS have increased risk of adverse pregnancy and neonatal complications. | It reviewed large number of eligible studies, and decreased heterogeneity by sensitivity analysis.  In addition, it eliminated metformin therapy and the most potential confounding variables of multiple pregnancies on PCOS patients. | Fail to provide the independent risk factor for indicating effect on the chance of developing adverse pregrance complications.  In addition, it was not possible to account for how the prevalence of pregnancy and neonatal complications changes follow the phenotypic variants of PCOS. |
| Hai-Feng Yu et al. | 2016 | 17816/123756 | GDM (RR: 2.78; 95% CI: 2.27-3.40);  PE (RR: 2.79; 95% CI: 2.29-3.38);  PIH (RR: 2.46; 95% CI: 1.95-3.09);  preterm delivery (RR: 1.52; 95% CI: 1.22-1.90);  cesarean delivery (RR: 1.25; 95% CI: 1.15-1.36);  miscarriage (RR: 2.87; 95% CI: 1.65-4.98);  hypoglycemia (RR: 2.85; 95% CI: 1.93-4.22);  perinatal death (RR: 1.83; 95% CI: 1.06-3.16). | PCOS in pregnancy is associated with a significantly increased risk of adverse pregnancy, fetal, and neonatal outcomes. | A large sample size and stratifing by study design, mean age, and pre-BMI. | The effect estimate in individual study with different adjusted factors.  Publication bias.  Individual data were not available, so it was impossible to perform more detailed and relevant analysis. |
| Mahnaz Bahri Khomami et al. | 2018 | 13549/210587 | miscarriage (OR: 1.59, 95% CI: 1.11-2.28);  GDM (OR: 2.89, 95% CI: 2.37‐3.54);  PIH (OR: 2.58, 95% CI: 1.95-3.41);  PE (OR: 1.87, 95% CI: 1.55-2.25);  induction of labour (OR: 2.55, 95% CI: 1.23‐5.30);  Caesarean section (OR: 1.39, 95% CI: 1.23‐1.57). | PCOS is associated with an increased risk of maternal pregnancy and delivery complications. The association of PCOS with the  outcomes is worsened in hyperandrogenic PCOS phenotypes, in specific geographic continents, and in the highest quality studies but disappears in assisted pregnancies. | Subgroup analyses were performed for a range of potential confounders, and meta‐regression explored the source of heterogeneity. | Lacking non‐English studies;  some studies had moderate to high risk of bias;  some studies have a small sample size;  differing PCOS definitions;  lack of definition or inconsistent reporting of obstetric outcomes;  lack or inconsistent reporting of ethnicity across included studies;  limited outcomes being reported according to BMI categories, spontaneous conception, pregnancies from ovulation induction, and multiple pregnancies;  lack of sufficient number of observations on the majority of confounding variables for meta‐regression;  and lack of data on perinatal depression and the impact of depression on pregnancy outcomes in women with and without PCOS. |
| Tingting Sha et al. | 2019 | 19679/45099 | Miscarriage (OR 1.41, 95% CI 1.04-1.91);  OHSS (OR 4.96, 95% CI 3.73-6.60);  GDM (OR 2.67, 95% CI 1.43-4.98);  PIH (OR 2.06, 95% CI 1.45-2.91);  preterm birth (OR 1.60, 95% CI 1.25-2.04);  LGA (OR 2.10, 95% CI 1.01-4.37). | Women with PCOS showed similar rates of clinical pregnancy, multiple pregnancy, ectopic pregnancy, small for gestational age and congenital malformations, and a higher live  birth rate, compared with women without PCOS. | It offered updating of evidence on a comprehensive range of obstetric and neonatal outcomes, some of which were not studied in the previous study.  It reviewed large number of eligible studies, and decreased heterogeneity by subgroup and sensitivity analyses.  Most of the eligible studies included in the meta-analysis were cohort studies of high quality. | Lack of prospective data to establish causation.  Some heterogeneity remained among the included studies.  The relatively small number of studies limited the statistical power and possibility of conducting further subgroup analyses.  There are some differences in treatment protocols or causes of infertility in the control group.  The current study was unable to perform further analysis to evaluate the influence of phenotypic variants of PCOS on the prevalence of pregnancy and neonatal complications. |
